# Loss of the Metastasis Suppressor NME1, But Not of Its Highly Related Isoform NME2, Induces a Hybrid Epithelial–Mesenchymal State in Cancer Cells

**DOI:** 10.3390/ijms22073718

**Published:** 2021-04-02

**Authors:** Anda Huna, Béatrice Nawrocki-Raby, Teresita Padilla-Benavides, Julie Gavard, Sylvie Coscoy, David Bernard, Mathieu Boissan

**Affiliations:** 1 Cancer Research Center of Lyon, INSERM U1052, CNRS UMR 5286, Léon Bérard Center, Lyon University, 69008 Lyon, France; anda.HUNA@lyon.unicancer.fr (A.H.); david.bernard@lyon.unicancer.fr (D.B.); 2 Université de Reims Champagne Ardenne, INSERM, P3Cell UMR-S 1250, SFR CAP-SANTE, 51097 Reims, France; beatrice.raby@univ-reims.fr; 3 Molecular Biology and Biochemistry Department, Wesleyan University, Middletown, CT 06459, USA; tpadillabena@wesleyan.edu; 4 Team SOAP, CRCINA, Inserm, CNRS, Université de Nantes, Université d’Angers, 44000 Nantes, France; julie.gavard@inserm.fr; 5 Integrated Center for Oncology, ICO, 44800 St. Herblain, France; 6 Institut Curie, Université PSL, Sorbonne Université, CNRS UMR168, Laboratoire Physico Chimie Curie, 75005 Paris, France; sylvie.Coscoy@curie.fr; 7 Equipe Labellisée «Ligue Contre le Cancer», 75006 Paris, France; 8 Sorbonne Université, Inserm, Centre de Recherche Saint-Antoine, CRSA, 75012 Paris, France; 9 Laboratory of Biochemistry and Hormonology, Tenon Hospital, AP-HP, 75020 Paris, France

**Keywords:** cancer, metastasis, epithelial–mesenchymal transition, epithelium

## Abstract

Epithelial–mesenchymal transition (EMT) is important for the initial steps of metastasis. Although it is well accepted that the nucleoside diphosphate kinase NME1 is a metastasis suppressor, its effect on EMT remains poorly documented, as does that of its closely related isoform, NME2. Here, by using gene silencing, inactivation and overexpression strategies in a variety of cellular models of cancer, we show that NME1 is a powerful inhibitor of EMT. Genetic manipulation of NME2, by contrast, had no effect on the EMT phenotype of cancer cells, indicating a specific function of NME1 in EMT regulation. Loss of NME1 in epithelial cancer cells resulted in a hybrid phenotype intermediate between epithelial and mesenchymal cells, which is known to be associated with cells with a highly metastatic character. Conversely, overexpression of NME1 in mesenchymal cancer cells resulted in a more epithelial phenotype. We found that NME1 expression was negatively associated with EMT markers in many human cancers and was reduced in human breast tumor cell lines with the aggressive ‘triple-negative’ phenotype when compared to human breast tumor cell lines positive for estrogen receptor. We show that NME1, but not NME2, is an inhibitor of essential concerted intracellular signaling pathways involved in inducing EMT, including the AKT and MAPK (ERK, p38, and JNK) pathways. Additionally, NME1 depletion considerably altered the distribution of E-cadherin, a gatekeeper of the epithelial phenotype, shifting it from the plasma membrane to the cytosol and resulting in less E-cadherin on the cell surface than in control cells. Functional aggregation and dispersion assays demonstrated that inactivation of *NME1* decreases E-cadherin-mediated cell–cell adhesion. We conclude that NME1, but not NME2, acts specifically to inhibit EMT and prevent the earliest stages of metastasis.

## 1. Introduction

Most deaths from cancer are due to metastases. Metastasis dissemination is a complex and highly inefficient process comprising tumor invasion into surrounding tissue at the primary tumor site, survival and arrest in the bloodstream, and progressive outgrowth at a site in a distant organ. At this secondary organ, the disseminated tumor cells must adapt to the new microenvironment and proliferate to generate macro-metastases, which can be detected in the clinic [1].

EMT, originally described as an integral part of morphogenesis in embryonic development, was later observed in the pathogenesis of several diseases including cancer progression. During EMT, epithelial cells are transcriptionally reprogrammed, resulting in decreased cell adhesion and enhanced migration and invasion [2]. EMT-inducing transcription factors (EMT-TFs; including SNAI1/2, ZEB1/2, TWIST1/2) are key regulators of cell–cell adhesion, cell polarity, cell motility and invasion during this process. These EMT-TFs are part of a complex network that represses the epithelial phenotype by inhibiting expression of epithelial proteins such as E-cadherin and that induces mesenchymal markers such as vimentin and N-cadherin [3,4]. Exogenous overexpression of many EMT-TFs enhances migration and/or invasion of cancer cells in vitro [5,6] and enhances metastasis in vivo [7,8]. Emerging evidence indicates that EMT-TFs can also upregulate various matrix degrading enzymes and induce the formation of invadopodia, both necessary to invade the local extracellular matrix [9]. This transcriptional reprogramming at EMT is induced by signaling pathways mediated by TGFβ, Wnt/β-catenin, Notch, Hedgehog, and tyrosine kinase receptors, including the receptors for EGF, HGF, and FGF. These pathways are activated by various dynamic stimuli from the local microenvironment, including growth factors and cytokines, hypoxia, metabolic and mechanical stresses, and matrix stiffness [2].

During EMT, the cells do not necessarily exist either in a purely epithelial or mesenchymal state, but rather in a hybrid epithelial–mesenchymal state [10]. It has become clear that EMT is not a simple binary switch from the epithelial to the mesenchymal phenotype, but comprises a spectrum of various cell states existing between the epithelial and mesenchymal phenotypes, depending on the activation of specific genetic programs and the properties of the microenvironment. The existence of hybrid epithelial–mesenchymal phenotypes was proposed initially after computational modelling of EMT regulatory networks [11,12,13,14]. Co-expression of epithelial and mesenchymal markers was reported in single cells of several cancer cell lines [15,16,17,18], in primary tumors, mouse models, and metastases during cancer progression [10,19]. Additionally, subpopulations of cells at various EMT stages and with different metastatic potentials were identified in primary mammary and skin tumors [10,20]. These studies point to the remarkably heterogeneous nature of EMT within tumors.

Nucleoside diphosphate kinases (NDPKs) are enzymes encoded by the *NME/NM23* gene family, which catalyze the synthesis of nucleoside triphosphates from their corresponding nucleoside diphosphates and ATP [21]. In humans, ten isoforms of the NME/NM23/NDPK family have been identified, among which the most abundant are the highly related proteins NME1 and NME2. Both NME1 and NME2 are 88% identical at the level of their amino acid sequences, and they have identical 3D structures; they are ubiquitous and are generally abundantly expressed. They are active as homo- and/or hetero-hexamers and are considered to be responsible for at least 80% of the NDPK activity in the cell. Both are mainly cytoplasmic enzymes, but they can also be found, at least transiently, associated with membranes and in nuclei. *NME1* has been the subject of considerable interest since its identification as the first metastasis suppressor gene [22,23]. Numerous studies have shown that loss of *NME1* expression correlates with metastasis and poor clinical prognosis in several types of human tumor, mainly those of epithelial origin [24]. The mechanistic basis for the anti-metastatic function of NME1 remains largely unknown, however. Moreover, the role of NME2 in metastasis is poorly documented.

Whereas the involvement of NME1 in tumor invasion and metastasis has been widely addressed, its role and the role of NME2 in EMT has hardly been studied. It is unclear, for example, whether depletion of NME1/NME2 might trigger a complete EMT, or a partial EMT, which is reported to be more effective than a full EMT in promoting metastasis [10,20]. Additionally, the relative contributions of NME1 and NME2 in this process remain poorly understood. This study aimed to investigate the different contributions of NME1 and NME2 to EMT, and the nature of this EMT, by inactivating or overexpressing the genes in a variety of cellular models.

## 2. Materials and Methods

### 2.1. Cell Culture

The MCF10DCIS.com cell line was purchased from Asterand (Detroit, MI, USA) and cultured in advanced DMEM/F12 medium containing 5% horse serum and 2 mM glutamine. MDA-MB-435 and MDA-MB-231T were kindly provided by P. S. Steeg and cultured in DMEM containing 10% fetal bovine serum (FBS) and 2 mM glutamine. MDCK-E-cadherin-GFP cells were a kind gift of W. J Nelson; they were cultured in DMEM containing 10% FBS and 0.4 mg/mL geneticin. Immortalized human cerebral microvascular endothelial cells (hCMEC/D3) were cultured as previously described [25]. BT-474, BT-549, HCC-1428, MDA-MB-468, PMC42 and ZR-75-1 cells were grown in RPMI-1640 medium containing 10% FBS and 100 U/mL penicillin and 100 µg/mL streptomycin (P/S). HCC-70, HCC-1143, HCC-1187, HCC-1599, HCC-1500, and HCC-1937 cells were grown in RPMI-1640 medium containing 10% FBS, P/S, 1.5 g/L sodium bicarbonate, 10 mM HEPES and 1 mM sodium pyruvate. T47D cells were grown in RPMI-1640 medium containing 10% FBS, P/S and 0.2 U/mL bovine insulin. BT-483 cells were grown in RPMI-1640 medium containing 20% FBS, P/S and 0.01 mg/mL bovine insulin. MDA-MB-231 cells were grown in DMEM-F12 containing 10% FBS and P/S. MCF-10A, MCF-10-2A, and 184B5 cells were grown in DMEM-F12 containing 5% horse serum, 20 ng/mL EGF, 100 ng/mL cholera toxin, 0.01 mg/mL insulin and 500 ng/mL hydrocortisone. MCF-12A and MCF-12F cells were grown in DMEM-F12 containing 5% horse serum, 20 ng/mL EGF, 100 ng/mL cholera toxin, 0.01 mg/mL insulin, 500 ng/mL hydrocortisone, 1.2 g/L sodium bicarbonate, 0.5 mM sodium pyruvate and 15 mM HEPES. HMEC and hTERT-HME1 cells were grown in Mammary Epithelial Cell Growth Medium BulletKit (Lonza, Basel, Switzerland). Hs578T and MDA-MB-361 cells were grown in DMEM containing with 10% FBS and P/S. BT-20 and MCF-7 cells were grown in MEM containing 10% FBS, 1.5 g/L sodium bicarbonate, 0.1 mM non-essential amino-acids and 1 mM sodium pyruvate. MDA-MB-157, MDA-MB-436, and MDA-MB-453 cells were grown in Leibovitz’s L-15 medium containing 10% FBS, P/S and 10 mM HEPES. MDA-MB-415 cells were grown in Leibovitz’s L-15 medium containing 15% FBS, P/S, 10 mM HEPES, 0.01 mg/mL insulin and 0.01 mg/mL glutathione. All cell lines were maintained at 37 °C in a humidified atmosphere with 5% CO_2_.

### 2.2. Antibodies

Selective rabbit polyclonal anti-NME1 and anti-NME2 antibodies were previously described [23]. Mouse monoclonal anti-N-cadherin antibody (clone 3B9) was purchased from ThermoFisher Scientific (Waltham, MA, USA). Mouse monoclonal anti-E-cadherin antibody (clone HECD-1) was purchased from ThermoFisher Scientific and used for Western blotting and flow cytometry analyses of MCF10DCIS.com cells. Rabbit monoclonal anti-E-cadherin antibody (clone 24E10) was purchased from Cell Signaling Technology, Inc. (Beverly, MA, USA) and used for Western blotting of MDCK cells. Rat monoclonal anti-E-cadherin antibody (clone DECMA-1) was purchased from ThermoFisher Scientific and used for flow cytometry of MDCK cells. Mouse monoclonal E-cadherin blocking antibody (clone SHE78-7) was purchased from ThermoFisher Scientific and used for cell dispersion assays [26]. Rabbit polyclonal anti-β-beta-catenin, mouse monoclonal anti-phospho-Thr^202^/Tyr^204^ ERK1/2 and rabbit polyclonal anti-ERK1/2 antibodies were purchased from Santa Cruz Biotechnology, Inc. (Dallas, TX, USA). Rabbit monoclonal anti-Slug and anti-ZEB1, rabbit polyclonal anti-phospho-Thr^183^/Tyr^185^ JNK and anti-JNK, rabbit monoclonal anti-phospho-Thr^180^/Tyr^182^ p38 and rabbit polyclonal anti-p38, rabbit polyclonal anti-phospho-Ser^473^ Akt and anti-Akt antibodies were purchased from Cell Signaling Technology, Inc. Mouse monoclonal anti-vimentin, anti-cytokeratin 18, and anti-α-tubulin (clone DM 1A) antibodies were obtained from Merck-Millipore (Burlington, MA, USA).

### 2.3. RNA Interference

All siRNA oligonucleotides were synthesized by ThermoFisher Scientific. The following siRNAs were used for *NME1*, the pool 5′-GGAUUCCGCCUUGUUGGUC-3′ and 5′-GGCUGUAGGAAAUCUAGUU-3′; *NME2*, the pool 5′-GGAUUGAUCAUUCUUUUAU-3′ and 5′-GCCUAUGGUUUAAGCCUGA-3′; irrelevant control siRNA: 5′-GGCUGUAGAAGCUAUAGUU-3′. MCF10DCIS.com and MDCK-E-cadherin-GFP cells were transfected with 100 nM control (scrambled siRNA) or specific siRNA duplex using Lipofectamine RNAiMax (ThermoFisher Scientific, Waltham, MA, USA). Protein depletion was verified by Western blotting analysis with selective rabbit polyclonal anti-NME1 and anti-NME2 antibodies and was maximal after 72 h of siRNA treatment.

### 2.4. CRISPR–Cas9 Gene Editing

CRISPR guides. Lentiviral plasmid guides targeting human *NME1* and *NME2* were generated in pLenti U6gRNA Cas9-GFP-Puro vector. They were purchased from Merck-Millipore as was the non-target guide (pLenti CRISPR-NT CONTROL). Two different guides were designed (https://www.milliporesigmabioinfo.com/bioinfo_tools/, accessed on 2 February 2016) for *NME1*: NME1-A [#HS0000009943, target sequence GACGGGCCGAGTCATGCTCGGG] and NME1-B [#HS0000009940, target sequence GAACACTACGTTGACCTGAAGG], and for *NME2*: NME2-A [#HS0000056847, target sequence TCATCGCCATCAAGCCGGACGG] and NME2-B [#NME2_0_76, target sequence AAGACCGACCATTCTTCCCTGG].

Lentiviral vectors production and MCF10DCIS.com cells transduction. These steps were performed by using the GIGA viral vectors platform (University of Liège, Belgium). Briefly, Lenti-X 293T cells (Takara Bio USA, Inc., Mountain View, CA, USA) were co-transfected either with pLenti U6gRNA *NME1*-Cas9-GFP-Puro, with pLenti U6gRNA *NME2*-Cas9-GFP-Puro, or with pLenti CRISPR-NT CONTROL and with the lentivirus packaging vectors pCgpV and pRSV-Rev and the envelope vector pCMV-VSV-G (all three from Cell Biolabs, Inc., San Diego, CA, USA). Lentiviral supernatants were collected 48 to 96 h post-transfection, filtrated and concentrated 100x by ultracentrifugation. Lentivirus stocks were titrated with qPCR Lentivirus Titration (Titer) Kit (Applied Biological Materials, Vancouver, Canada) and used to transduce cells. After 72 h, cells were selected with 2 µg/mL puromycin (InvivoGen, San Diego, CA, USA). Then, cells expressing GFP were isolated and cloned by FACS on a FACSaria III 4L sorter (BD Biosciences, San Jose, CA, USA). Each clone was tested by Western blotting and immunofluorescence analysis. Clones that were negative for NME1 or NME2 expression were selected for further experiments.

Sequencing. Selected clones were analyzed by miSeq in order to confirm mutations in *NME1* or *NME2*-coding sequences. DNA was extracted from cell pellets by using the Maxwell^®^ 16 Blood DNA Purification Kit. Primers flanking the CRISPR–Cas9 target sites were designed with Primer3 based on the UCSC hg19 human reference genome. Nextera XT adapter overhangs sequences and primers sequences are given in Table S1. For all clones, amplicons were generated for the four targeted regions (NME1A, NME1B, NME2A, and NME2B) by using Q5 High-Fidelity DNA Polymerase (New England Biolabs, Hitchin, UK). PCR1 products were purified with AMPure beads. Illumina sequencing adapters and dual index barcodes were added to the amplicon target libraries with only 8 cycles of PCR by using the Kapa Hifi HotStart ready mix (as described in the Illumina 16S sample preparation guide). Different combinations of Nextera XT index were used for each sample. PCR2 products were then purified with AMPure beads, quantified by using the picogreen dsDNA quantitation assay, adjusted to 7 ng/µL and then pooled. Before high-throughput sequencing, the final pools were quantified by qPCR (KAPA SYBR FAST kit (ABI Prism). Final libraries were spiked (8%) into a Miseq run 300 cycles v2 (Read1: 156 cy, Read2:160 cy, index1: 8cy, index2: 8cy).

Sequence analysis. Raw reads were demultiplexed and adapter-trimmed using Illumina bcl2fastq. Analysis of the sequencing data was performed using CRISPResso v1.0.2 (https://www.ncbi.nlm.nih.gov/pubmed/27404874, accessed on 2 September 2017) by comparing the sequence of each amplicon of each clone to the corresponding region in the UCSC hg38 reference the human genome. Reads containing insertions and/or deletions (indel) with respect to the reference amplicon sequence were identified and considered as edited whereas reads only containing substitutions were conservatively considered as not edited (CRISPResso options: —ignore_substitutions and no guide provided). The region of the amplicon containing coding sequences was also provided to identify out-of-frame indels (CRISPResso -c option). Analyses performed in CRISPResso with alternative options (counting modifications in a window of 7 nucleotides around the predicted cutting site and with or without ignoring substitutions) gave similar results. Sequencing results were also checked visually in the Integrative Genome Viewer after alignment directly to the entire UCSC hg38 Human reference genome with BWA v0.7.5a (BWA mem algorithm).

### 2.5. DNA Extraction, PCR Amplification, DNA Typing of Human Breast Cell Lines

Genomic DNA was extracted from the cell line pellets by using the Quiamp DNA mini kit^®^, from Qiagen (Hilden, Germany), according to the manufacturer’s recommendations. Short tandem repeat analysis was performed using the Powerplex 16HS^®^ kit from Promega (Madison, WI, USA) according to the supplier’s instructions. PCR-based multiplex amplification was used to amplify 15 short tandem repeats (Penta E, D18S51, D21S11, THO1, D3S1358, FGA, TPOX, D8S1179, vWA, and a sex determination marker). PCR was performed in a 25 µL reaction volume, including 0.5 ng of genomic DNA, 5 µL master mix and 2.5 µL Primer pair mix and completing the volume with ddH_2_O. PCR thermal cycling conditions were: pre-incubation for 2 min at 96 °C, followed by 10 cycles at 94 °C for 30 s, 60 °C for 30 s, 70 °C for 45 s; and 22 cycles at 94 °C for 30 s, 60 °C for 30 s, 70 °C for 45 s with a final extension at 60 °C for 30 min. All amplifications were done on GeneAmp PCR 9700 thermal cycler (ThermoFisher Scientific). PCR amplicons were separated by electrophoresis and were detected with an ABI 3100 Genetic Analyzer (ThermoFisher Scientific) using 1 µL PCR product or allelic ladder mixed with 18.5 µL Hi-Di Formamide and 0.5 µL Internal Lane Standard 600. The loading mixture was denatured at 95 °C for 3 min followed by chilling on ice immediately. Short tandem repeat genotyping was performed by using GeneMapper 5.0 (ThermoFisher Scientific).

### 2.6. RT-qPCR Analysis of Human Mammary Cell Lines

Samples of the human mammary cell lines were analyzed by RT-qPCR. The conditions for total RNA extraction, cDNA synthesis and PCR reaction were described previously [27]. Quantitative values were obtained from the cycle number (Ct value) by using the ABI Prism 7900HT Sequence Detection System and PE Biosystems analysis software according to the manufacturer’s instruction (Perkin-Elmer Applied Biosystems, Foster City, CA, USA). Data from each sample were normalized to the amount of *TBP* transcript. *TBP*, which encodes the TATA box-binding protein, was selected as an endogenous control due to the moderate level of its transcripts and the absence of known *TBP* retro-pseudogenes, which might lead to co-amplification of contaminating genomic DNA and thus interfere with RT-PCR transcripts, despite the use of primers in separate exons. The data, expressed as N-fold differences in target gene expression relative to the *TBP* gene and termed ‘N_target_’, were determined as N_target_ = 2^ΔCtsample^, where the ΔCt value of the sample was determined by subtracting the average Ct value of the target gene from the average Ct value of *TBP*. mRNA levels in the human mammary cell lines were also normalized to obtain a ‘basal’ mRNA level (smallest amount of mRNA quantifiable (Ct = 35 with 2.5 ng cDNA)) equal to 1. Primers for *NME1* (upper primer, 5’-ATCAAACCAGATGGGGTCCAG-3’; lower primer, 5’-AGAAGATCTTCGGAAGCTTGCAT-3’) and *TBP* (upper primer, 5’-TGCACAGGAGCCAAGAGTGAA-3′; lower primer, 5’-CACATCACAGCTCCCCACCA-3′) were selected by using the Oligo 6.0 program (National Biosciences, Plymouth, MN, USA). Normal-like human mammary cell lines were the following: 184B5, HMEC, hTert HME1, MCF-10-2A, MCF-10A, MCF-12A, and MCF-12F. Estrogen-receptor-positive human mammary tumor cell lines were the following: BT474, BT483, HCC-1428, HCC1500, MCF7, MDA-MB-361, MDA-MB-415, PMC42, T47D, and ZR75-1. Triple-negative human mammary tumor cell lines (negative for estrogen receptor, progesterone receptor, and HER2) were the following: BT20, BT549, HCC-1143, HCC-1187, HCC-1599, HCC-1937, HCC-70, Hs 578T, MDA-MB-157, MDA-MB-231, MDA-MB-453, MDA-MB-436, and MDA-MB-468.

### 2.7. RT-qPCR of Breast Tumor Cell Lines Genetically Modified for NME1 and NME2

Total RNA was isolated by phenol-chloroform extraction. The Maxima First cDNA Synthesis Kit (ThermoFisher Scientific) was used to synthesize cDNA from 4 µg of RNA according to the manufacturer’s instructions. TaqMan qPCR analyses were carried out on a FX96 Thermocycler (BioRad, Hercules, CA, USA). The PCR mixture contained TaqMan mix (Roche, Basel, Switzerland), 200 nM of primers, the Universal Probe Library probe (100 µM) for the gene of interest (TaqMan Gene Expression Assays [Primers/probe]; ThermoFisher Scientific), and 1.67 μL cDNA template. Reactions were performed in technical duplicates. The relative amount of mRNA was calculated by using the comparative Ct (ΔΔCt) method and by normalizing to the amount of mRNA for the housekeeping genes *PGK1* and *TBP*. The PCR primers were the following: *TWIST1* (left primer, 5′-GGCTCAGCTACGCCTTCTC-3′; right primer, 5′-CCTTCTCTGGAAACAATGACATCT-3′), *TWIST2* (left primer, 5′-CATGTCCGCCTCCCACTA-3′; right primer, 5′-GCATCATTCAGAATCTCCTCCT-3′), *SNAI1* (left primer, 5′-GCTGCAGGACTCTAATCCAGA-3′; right primer, 5′-ATCTCCGGAGGTGGGATG-3′), *SNAI2* (left primer, 5′-TGGTTGCTTCAAGGACACAT-3′; right primer, 5′-GTTGCAGTGAGGGCAAGAA-3′), *ZEB1* (left primer, 5′-AACTGCTGGGAGGATGACAC-3′; right primer, 5′-TCCTGCTTCATCTGCCTGA-3′), ZEB2 (left primer, 5′-AAGCCAGGGACAGATCAGC-3′; right primer, 5′-AAGCCAGGGACAGATCAGC-3′), TBP (left primer, 5‘-CCCATGACTCCCATGACC-3′; right primer, 5′-TTTACAACCAAGATTCACTGTGG-3′), and *PGK1* (left primer, 5′-CAGCTGCTGGGTCTGTCAT-3′; right primer, 5′-GCTGGCTCGGCTTTAACC-3′).

### 2.8. E-Cadherin Cell Surface Expression

The surface of cells genetically manipulated for *NME1* and *NME2* was labeled with an antibody against E-cadherin. Briefly, the different cell populations were blocked with 3% BSA in PBS/EDTA for 1 h, incubated with primary antibody for 30 min, washed three times with PBS, incubated with the appropriate AlexaFluor-488-and PE-conjugated secondary antibodies, and washed an additional three times with PBS. To ensure surface labeling, all solutions were ice-cold and the cells were kept on ice during all incubation steps. Following labeling, cells were fixed in 2% paraformaldehyde and kept in the dark until analysis. Control cells were stained in parallel with secondary antibody to estimate background. Labelling was quantified by using a Gallios Flow Cytometer (Beckman-Coulter, Brea, CA, USA). The mean fluorescence intensity for control cells (secondary antibody staining only) was subtracted from the mean fluorescence intensity for each cell line population, and the data were presented as a percentage of expression relative to the scrambled siRNA or the non-targeting CRISPR–Cas9.

### 2.9. Quantification of E-Cadherin Fragments

Gel bands were quantified by using the ‘Analyze Gels’ module in ImageJ (http://imageJ.nih.gov/ij/index.html, accessed on 2 October 2020). For each band, the intensity of the cleavage products between 70 and 25 kDa was measured, and the sum of the intensities of the cleavage products was normalized to the sum of the intensities of endogenous E-cadherin and E-cadherin-GFP bands. For each gel, the intensities of the bands were normalized to the mean of the control values. To avoid saturation of the signal, when necessary, a different exposure time was used for quantification of the non-degraded bands and of the cleavage products.

### 2.10. Western Blotting Analysis

Cells were lysed in 50 mM Tris-HCl pH 7.5, 137 mM NaCl, 10 mM MgCl2, 10% glycerol and 1% Triton-X100 with protease inhibitors. Proteins from cell extracts were separated on 10% SDS polyacrylamide gels and transferred onto nitrocellulose membranes, which were then incubated with primary antibodies. Blots were revealed with appropriate peroxidase-coupled secondary antibodies and chemiluminescent ECL Plus substrate (GE Healthcare, Chicago, IL, USA).

### 2.11. Cell Morphology and Line Scan Profiling

Images of cells, MCF10DCIS.com, MDA-MB-435, MDA-MB-231T, and MDCK-E-cadherin-GFP genetically modified for *NME1* and *NME2*, were acquired with an inverted Olympus IX83 microscope, 20X dry objective, Cellsens Imaging software (Olympus, Japan), and intensity profiles in cells were measured with Fiji.

### 2.12. Permeability Assay

FITC-dextran transcellular passage was estimated on 3-day-old hCMEC/D3 monolayers cultured on 3 µm pore collagen-coated PTFE membranes (Corning, Corning, NY, USA) as previously described [28] and analyzed with a fluorescence plate reader (PerkinElmer Inc., Wellesley, MA, USA).

### 2.13. Cell Adhesion Assay

Confluent monolayers of cells were washed with ice-cold PBS, and detached from the plates by incubation with PBS without Ca^2+^ supplemented with 0.6 U/mL of dispase I (MP Biomedicals, Irvine, CA, USA) for 35 min at 37 °C. The dispase solution was removed, and replaced by 200 µL of PBS. The cells were then mechanically separated by pipetting up and down five times in a 200 µL pipette. The resulting aggregates were observed by light microscopy using the 10X objective (Echo Rebel Microscope, San Diego, CA, USA). The size of aggregates was measured using the Fiji software [29] (aggregates <200 µm^2^ were excluded from the quantification [30]).

### 2.14. Cell Dispersion Assay

Cells were seeded in 12-well culture plates in the presence of an E-cadherin blocking antibody at a final concentration of 1 µg/mL or of a mouse IgG isotype control. Forty-eight hours after seeding, phase-contrast images were taken at a 20-fold magnification using the EVOS XL core cell imaging system (ThermoFisher Scientific). Spatial distribution of cells was analyzed by a plugin developed at INSERM Unit 1250 for ImageJ software (http://imageJ.nih.gov/ij/index.html, accessed on 15 January 2021), which calculates Voronoi’s partition, Delaunay’s graph and minimum spanning tree (MST) as previously described [26].

### 2.15. METABRIC and TCGA Databases

Gene expression data were extracted from cBioPortal for Cancer Genomics (https://www.cbioportal.org/, accessed on 2 August 2020), which allows the visualization, analysis, and downloading of large-scale cancer genomics data sets [31,32], by specifically focusing on the METABRIC (Molecular Taxonomy of Breast Cancer International Consortium) [33,34] and TCGA (The Cancer Genome Atlas) databases. EMT signature was calculated by using the methodology defined previously [35]. In brief, it corresponds to the sum of expression of genes increased in EMT minus the sum of the genes decreased in EMT.

### 2.16. Statistical Analysis

Statistical analyses were performed using Student’s *t*-test with the Prism version 5.0 software (GraphPad Software, La Jolla, CA, USA). All tests were two sided. *p*-values < 0.05 were considered significant.

## 3. Results

### 3.1. Depletion of NME1 from Epithelial Breast Cancer Cells Induces an Incomplete EMT

**Figure 1:**
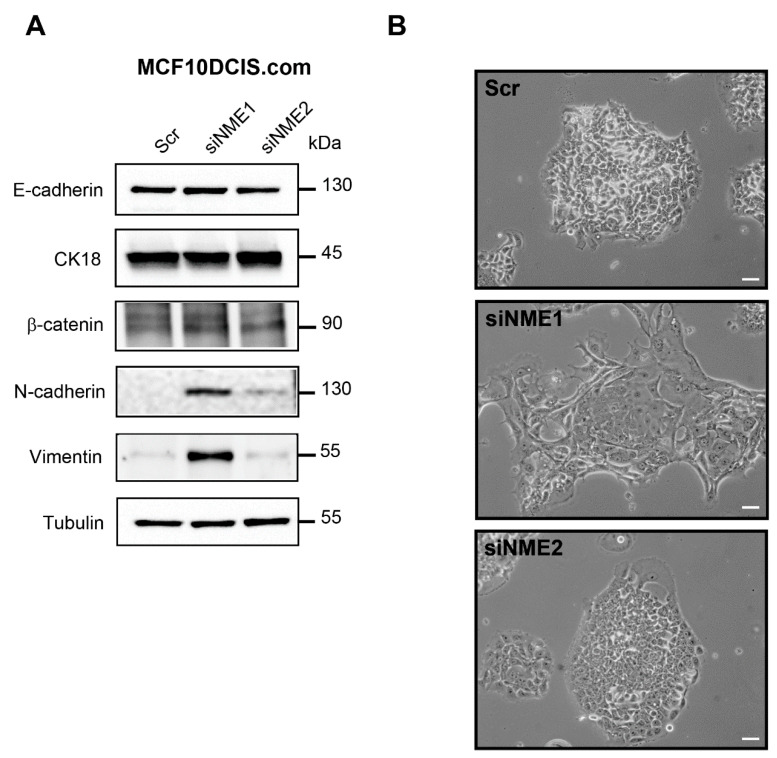
Depletion of *NME1* from epithelial breast cancer cells induces an incomplete EMT. (**A**) MCF10DCIS.com human breast carcinoma cells, which have an epithelial-like phenotype, were treated with siRNAs to silence *NME1* (siNME1) or *NME2* (siNME2) or with a scrambled siRNA (Scr) as a control. Cell lysates were analyzed by Western blotting with antibodies against the indicated proteins. Equal loading was verified by Western blotting for tubulin. Molecular weights are indicated in kDa. (**B**) Phase-contrast microscopy of control and silenced cells as in (**A**) 72 h post-transfection. Scale bar, 20 μm.

As a first step to investigate the contributions of NME1 and NME2 to EMT, we depleted a human breast carcinoma cell line, MCF10DCIS.com [36], which has an epithelial-like phenotype, specifically of *NME1* or *NME2* by expressing siRNAs and then measured expression of EMT-related proteins including E-cadherin, cytokeratin 18, β-catenin, N-cadherin, and vimentin by Western blotting (Figure 1). Western blotting confirmed the effective siRNA-mediated knockdown of *NME1* and *NME2* (Supplemental Figure S1A). Expression of the epithelial markers, E-cadherin, cytokeratin 18, and β-catenin was unchanged, whereas the mesenchymal markers N-cadherin and vimentin were strongly increased in *NME1*-depleted cells when compared to control cells (Figure 1A). In *NME2*-depleted cells, E-cadherin, cytokeratin 18, and β-catenin levels were similar to those observed in *NME1*-depleted and control cells; by contrast, *NME2* knockdown did not induce expression of N-cadherin and vimentin (Figure 1A). The N-cadherin protein has a predicted molecular weight of 100 kDa; however, it is extensively glycosylated and has been shown to migrate in SDS-PAGE with an apparent size of 125–135 kDa [37,38]. In MCF10DCIS.com cells, we observed only one band at 130 kDa, indicating that all the N-cadherin is glycosylated in these cells. We observed the morphology of the depleted and control MCF10DCIS.com cells by phase-contrast microscopy and found that whereas the control cells were typically polygonal and formed an epithelium-like monolayer many of the cells in which *NME1* was silenced had lost their epithelial properties and adopted a spindle-shaped mesenchymal morphology (Figure 1B). Cells in which *NME2* was silenced, by contrast, retained a typical epithelial morphology similar to that of the control cells (Figure 1B). Thus, *NME1*-depleted MCF10DCIS.com cells acquire mesenchymal traits but retain epithelial markers, showing that the specific loss of *NME1*, but not of *NME2*, induces an incomplete EMT. 

### 3.2. Inactivation of NME1 by CRISPR–Cas9 Gene Editing Reinforces EMT

To obtain cell lines in which the *NME1* or *NME2* genes were completely and stably inactivated, we used CRISPR–Cas9 gene editing in MCF10DCIS.com cells with two independent guide RNAs specific for the *NME1* gene, NME1(#A) and NME1(#B), and two for the *NME2* gene, NME2(#A) and NME2(#B). To provide a control cell line for experiments with the *NME1*- and *NME2*-ablated cells, we subjected MCF10DCIS.com cells to the CRISPR–Cas9 procedure in the presence of a non-targeting control guide RNA [Non-targeting (NT) cells]. The NT cells had an epithelial morphology similar to that of MCF10DCIS.com cells treated with a control, scrambled siRNA. Genetic and Western blotting analyses confirmed the total absence of NME1 and NME2 in their respective *NME1*- and *NME2*-ablated cells (Supplemental Figures S1B and S2 and Table S1). Selective loss of NME1 or NME2 did not alter the protein level of the other isoform, showing that NME1 and NME2 expression are regulated independently (Supplemental Figure S1B). 

**Figure 2:**
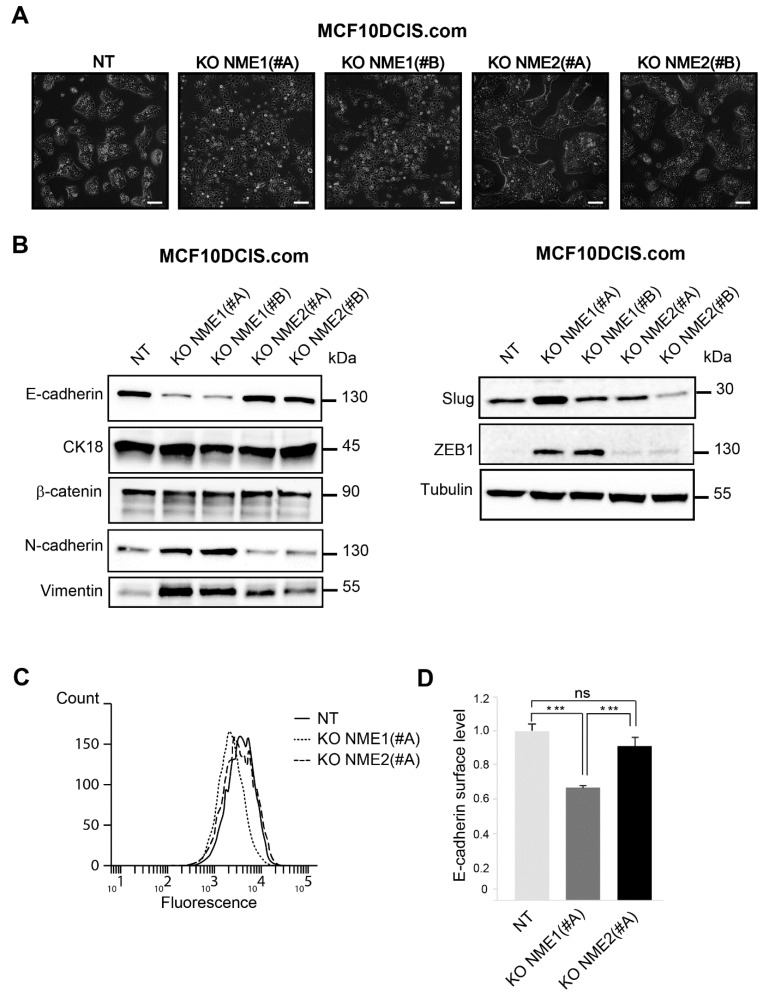
Inactivation of NME1 by CRISPR–Cas9 gene editing reinforces EMT. (**A**) Phase-contrast microscopy of control non-targeting (NT) and *NME1* or *NME2* knockout (KO) MCF10DCIS.com treated with two different guide RNAs (#A and #B) for each gene, as indicated. Scale bar, 50 μm. (**B**) Lysates of NT and KO cells were analyzed by Western blotting with antibodies against the indicated proteins. Equal loading was verified by Western blotting for tubulin. Molecular weights are indicated in kDa. (**C**) NT and KO cells were analyzed by FACS for cell surface E-cadherin levels. (**D**) Quantification of FACS analysis as in (**C**). Three independent experiments were performed. Data are expressed as means ± SEM. *** *p* < 0.001, ns: not significant.

*NME1*-ablated cells tended to scatter, form elongated protrusions, and loose intercellular adhesion, unlike NT cells and *NME2*-ablated cells, which formed well-delimited clusters (Figure 2A). When analyzed for EMT-related proteins, including epithelial and mesenchymal markers and EMT-TFs, *NME1*-ablated cells, whether treated with the NME1(#A) or NME1(#B) guide, displayed a marked EMT character with low levels of E-cadherin protein, no modification of cytokeratin 18 and β-catenin levels, and increased levels of N-cadherin and vimentin when compared to the NT control (Figure 2B). There was striking upregulation of EMT-TF protein ZEB1 in *NME1*-ablated cells treated with either the NME1(#A) or the NME1(#B) guide, whereas the EMT-TF Slug was upregulated only in cells that had been treated with the NME1(#A) guide (Figure 2B); this latter effect might be explained by differences between the two clones of NME1-inactivated cells. In *NME2*-ablated cells, the levels of all of these proteins were similar to those in NT cells (Figure 2B). A significant reduction in the levels of E-cadherin on the cell surface, measured by flow cytometry, was also observed in *NME1*-ablated cells when compared to *NME2*-ablated cells and NT controls (Figure 2C,D). Together, these data show that the absence of *NME1*, but not of *NME2*, is sufficient to induce strongly markers of EMT. The persistence of the epithelial markers cytokeratin 18 and β-catenin in *NME1*-ablated cells at levels similar to those in control cells, however, indicates that the EMT phenotype remains incomplete in the absence of *NME1*.

We measured the levels of EMT-TF mRNAs in MCF10DCIS.com cells that were genetically modified for *NME1* and *NME2* (Supplemental Figure S3A). *NME1*-ablated cells, whether treated with the NME1(#A) or NME1(#B) guide, had specifically upregulated levels of *ZEB1* and *SNAI2* mRNA when compared to the NT control. Additionally, levels of *TWIST2* and *ZEB2* mRNAs tended to be higher in *NME1*-ablated cells, although these levels were not statistically different from those in the NT control. Levels of *TWIST1* mRNA were unchanged in *NME1*-ablated cells. Levels of *SNAI1* mRNA were downregulated in *NME1*-ablated cells when compared to the NT control. In the *NME2*-ablated cells, by contrast, whether treated with the NME2(#A) or NME2(#B) guide, the levels of all the EMT-TF mRNAs were similar to those in NT cells, with the exception of *SNAI1* mRNA, whose levels were decreased.

### 3.3. E-cadherin Distribution Is Altered in NME1-Depleted Cells

**Figure 3:**
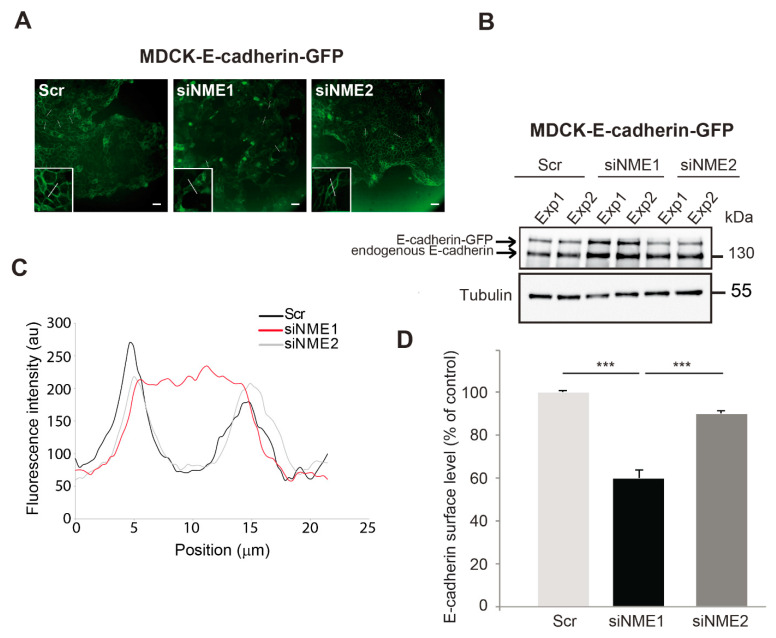
E-cadherin distribution is altered in *NME1*-depleted epithelial cells. (**A**) GFP fluorescence of control (Scr) MDCK epithelial cells stably overexpressing E-cadherin-GFP (MDCK E-cadherin-GFP) and the same cells treated with siRNAs to silence *NME1* (siNME1) or *NME2* (siNME2) 72h post-transfection. White lines indicate typical regions used for the line scan profiling shown in (**C**). Scale bar, 25 μm. (**B**) Lysates of cells as in (**A**) were analyzed by Western blotting with an antibody against E-cadherin. The results of two independent experiments (Exp1 and Exp2) are shown. Equal loading was verified by Western blotting for tubulin. Molecular weights are indicated in kDa. (**C**) Line scan profiling of the control, siNME1, and siNME2 MDCK cell populations. (**D**) Cells as in (**A**) were analyzed by FACS for cell surface E-cadherin levels and quantified. Three independent experiments were performed. Data are expressed as means ± SEM. *** *p* < 0.001.

The experiments described above suggest the existence of two distinct intermediate EMT states, depending on the intensity of *NME1* depletion: upon depletion of *NME1* by RNAi, E-cadherin protein levels were unaltered whereas only upon inactivation of the *NME1* gene by CRISPR–Cas9 were they decreased. To investigate further this notion of a partial EMT phenotype induced by *NME1* depletion, we assessed the effects of depletion on the subcellular distribution of E-cadherin in an epithelial cell line, MDCK, expressing E-cadherin–GFP, in which E-cadherin organization during junction formation is well characterized [39]. We focused on the behavior of cells at early stages of junction formation, in line with the experiments presented on MCF10DCIS.com cells (Figure 3). Western blotting confirmed the effective depletion of *NME1* and *NME2* by siRNA-mediated knockdown in this cell line (Supplemental Figure S1C). As in our experiments with MCF10DCIS.com cells, the MDCK-E-cadherin–GFP cells were plated at a sub-confluent state (low density) one day before transfection. At the time of transfection, these cells had already formed strong cell–cell contacts in well-delimited clusters. Upon depletion of *NME1*, but not of *NME2*, we observed a progressive loss of cohesion within the multicellular islets, reflecting partial loss of contacts between cells (Figure 3A). The level of endogenous E-cadherin and E-cadherin-GFP was slightly higher in cells depleted of *NME1* than in cells depleted of *NME2* or in the control cells transfected with a scrambled RNAi sequence (Figure 3B). Consistent with the loss of intercellular contacts seen by microscopy, the distribution of E-cadherin was strongly altered in *NME1*-depleted cells, shifting from the plasma membrane to the cytosol and resulting in less E-cadherin on the cell surface, as assessed by line scan profiling (Figure 3C) and quantification of cell surface E-cadherin by FACS (Figure 3D). By contrast, the distribution of E-cadherin was not significantly affected by depletion of *NME2* from these cells. Thus, early in the formation of an epithelium, the distribution of E-cadherin is specifically altered by *NME1*-depletion of MDCK-E-cadherin-GFP cells, shifting from the plasma membrane to the cytosol as is characteristic of EMT. This is consistent with the phenotype described above for MCF10DCIS.com cells. These data indicate that loss of *NME1* considerably alters the distribution of E-cadherin at the onset of junction formation by epithelial cells.

Associated with the altered E-cadherin staining in *NME1*-depleted MDCK cells, total protein analysis by Western blotting with an anti-E-cadherin antibody revealed increased levels of different cleaved fragments of E-cadherin of 70–25 kDa in *NME1*-depleted cells, when compared to control or *NME2*-depleted MDCK cells (Supplemental Figure S4A). These sizes are consistent with the different cleaved fragments of E-cadherin, known to decrease cell–cell aggregation and increase migration and invasion [40]. E-cadherin cleavage is mediated by γ-secretases, which are members of the ADAM family of proteases; specifically, ADAM10 is centrally involved. We found that the protein levels of ADAM10 in *NME1*-depleted cells were much increased when compared to control cells (Supplemental Figure S4B). 

We also measured the protein levels of ADAM10 in tumor cells genetically modified for *NME1* or *NME2* (Supplemental Figure S4C,D). *NME1*-ablated MCF10DCIS.com cells, whether treated with the NME1(#A) or NME1(#B) guide, displayed increased levels of ADAM10 when compared to the NT control (Supplemental Figure S4C). In *NME2*-ablated MCF10DCIS.com cells, the levels of ADAM10 were similar to those observed in NT cells (Supplemental Figure S4C). By contrast, in MDA-MB-231T cells, overexpression of *NME1* reduced levels of ADAM10 (Supplemental Figure S4D). Based on these findings, we looked at the levels of *ADAM10* mRNA in the Cancer Genome Atlas (TCGA) human transcriptome database (Supplemental Figure S5 and Table S2). *NME1* mRNA levels were strongly negatively associated with mRNA levels of *ADAM10* in fifteen human cancer types (Table S2). Furthermore, a negative association between NME1 and ADAM10 protein levels was observed in 74 samples of breast invasive carcinoma in the TCGA database by mass spectrometry (Supplemental Figure S5A). Strong positive associations were observed between *NME1* mRNA levels and NME1 protein abundance (Supplemental Figure S5B) and also between *ADAM10* mRNA levels and ADAM10 protein abundance (Supplemental Figure S5C), suggesting that NME1 and ADAM10 gene expression are regulated mainly at the transcriptional level. These data reveal low NME1 expression associated with high ADAM10 expression is a common trait in human clinical tumor samples. 

### 3.4. Stable Overexpression of NME1 Reverses EMT in Mesenchymal Breast Cancer Cells

**Figure 4:**
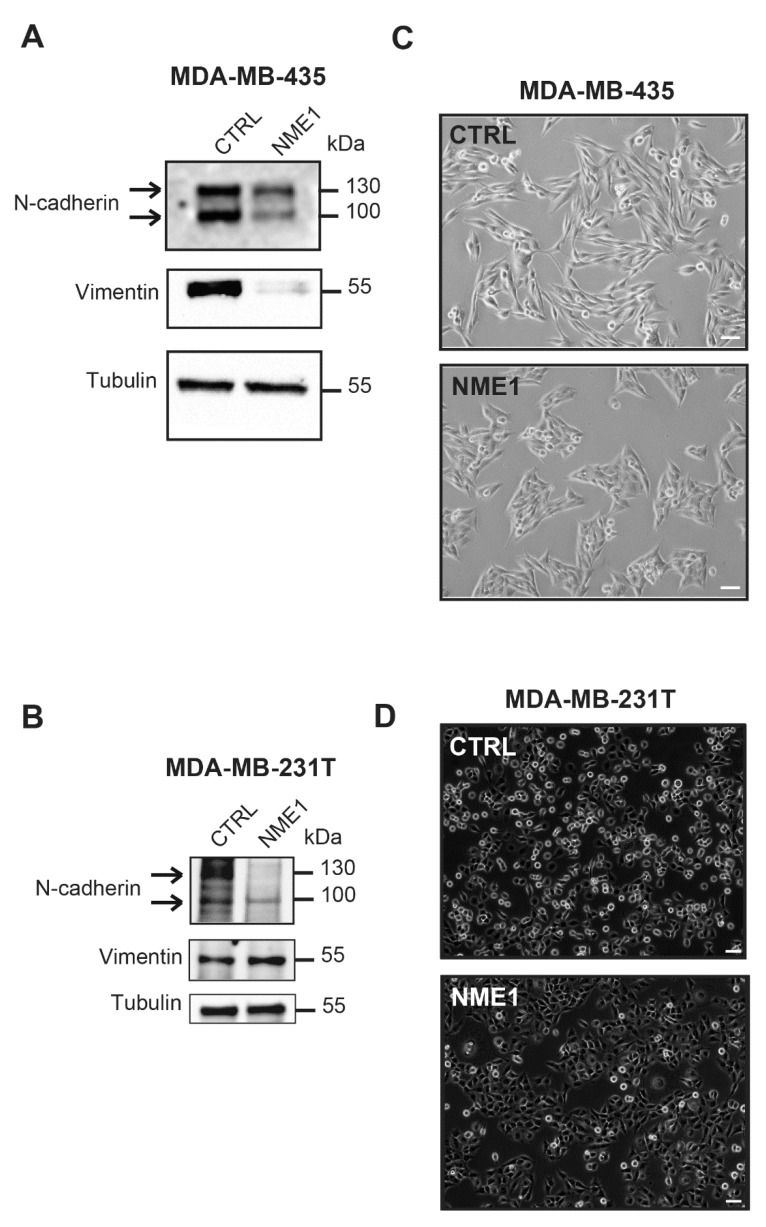
Stable overexpression of *NME1* reverses EMT in mesenchymal breast cancer cells. (**A**) Lysates of MDA-MB-435 human breast carcinoma cells stably transfected with the empty control vector (CTRL) or with a vector expressing *NME1* (NME1) were analyzed by Western blotting with antibodies against the indicated proteins. Equal loading was verified by Western blotting for tubulin. Molecular weights are indicated in kDa. (**B**) Lysates of MDA-MB-231T human breast carcinoma cells stably transfected with the empty control vector (CTRL) or *NME1*-encoding vector (NME1) and analyzed as in (**A**). (**C**) Phase-contrast microscopy of MDA-MB-435 cells overexpressing *NME1*. Scale bar, 50 μm. (**D**) Phase-contrast microscopy of MDA-MB-231T cells overexpressing *NME1*. Scale bar, 50 μm.

Since depletion of *NME1* from epithelial cancer cells induces an EMT phenotype, we wondered whether, conversely, overexpression of *NME1* in mesenchymal cancer cells might restore an epithelial phenotype. To test this, we stably overexpressed *NME1* in two highly invasive and metastatic human breast carcinoma cell lines, MDA-MB-435 and MDA-MB-231T, to determine its effects on the EMT phenotype. Neither cell line expresses E-cadherin [26] and cytokeratin 18 levels are undetectable in both cell lines [41,42]. Western blotting confirmed the strong overexpression of *NME1* in these cells (Supplemental Figure S1D,E). In MDA-MB-435 cells, overexpression of *NME1* strongly reduced levels of N-cadherin and vimentin, two markers of the invasive, mesenchymal phenotype (Figure 4A), whereas in MDA-MB-231T cells, it reduced N-cadherin protein levels but did not change the levels of vimentin (Figure 4B). In both MDA-MB-435 and MDA-MB-231T cells, glycosylated and unglycosylated forms of N-cadherin were seen as bands of 130 and 100 kDa on the Western blots. Overexpression of *NME1* did not change NME2 protein levels (Supplemental Figure S1D,E). Both MDA-MB-435 and MDA-MB-231T cells overexpressing *NME1* tended to form clusters whereas control cells transfected with the empty-vector were more scattered (Figure 4C,D) and the control MDA-MB-435 cells, furthermore, had a spindle-shaped morphology (Figure 4C). These findings indicate that overexpression of *NME1* prevents an EMT-like phenotype. Thus, whereas loss of *NME1* induces an EMT-like phenotype, *NME1* overexpression, conversely, inhibits it. 

We measured EMT-TF mRNA levels in the MDA-MB-435 and MDA-MB-231T cells overexpressing *NME1* (Supplemental Figure S3B,C). In MDA-MB-435 cells, overexpression of *NME1* reduced *SNAI1* and *SNAI2* mRNA levels two-fold, whereas levels of *ZEB1*, *ZEB2*, and *TWIST1* mRNAs remained unchanged when compared to the control condition; *TWIST2* was undetectable in these cells (Supplemental Figure S3B). In MDA-MB-231T cells, overexpression of *NME1* reduced *ZEB2* mRNA levels two-fold, whereas levels of *SNAI1*, *SNAI2*, and *ZEB1* mRNAs remained unchanged when compared to the control condition; both *TWIST1* and *TWIST*2 were undetectable in these cells (Supplemental Figure S3C).

### 3.5. Inactivation of NME1 Decreases E-Cadherin-Mediated Cell–Cell Adhesion

**Figure 5:**
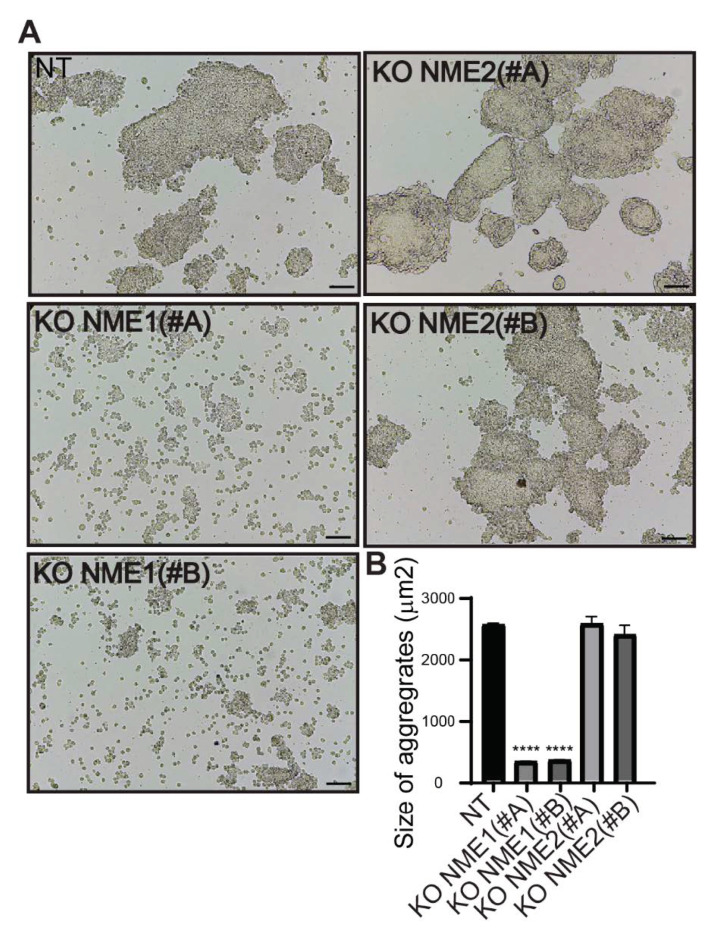
Inactivation of NME1 decreases cell–cell adhesion. (**A**) Representative light microscopy images showing dispase aggregation assays of MCF10DCIS.com cells in which *NME1* or *NME2* was inactivated. Scale bar, 100 µm. (**B**) The size of the aggregates was quantified as the area of their horizontal projections. Values shown are the means of three independent biological replicates ± SEM. **** *p* < 0.0001.

To investigate the functional consequences of genetically modifying expression of *NME1* and *NME2* in MCF10DCIS.com breast carcinoma cells, we studied their cell–cell adhesion properties. Deletion of *NME1* led to a decrease in the adhesive properties of these cells: cells treated with either the NME1(#A) or NME1(#B) guide no longer formed aggregates upon treatment with dispase, but were readily dispersed (Figure 5A). Deletion of *NME2*, by contrast, had no effect on the formation of cell aggregates upon treatment with dispase; like the control NT cells, cell aggregates remained after treatment (Figure 5A). On average, the aggregates formed by NT and *NME2*-ablated cells were significantly larger (on average 2500 µm^2^) than those formed by the *NME1*-ablated cells (200–400 µm^2^; Figure 5B).

**Figure 6:**
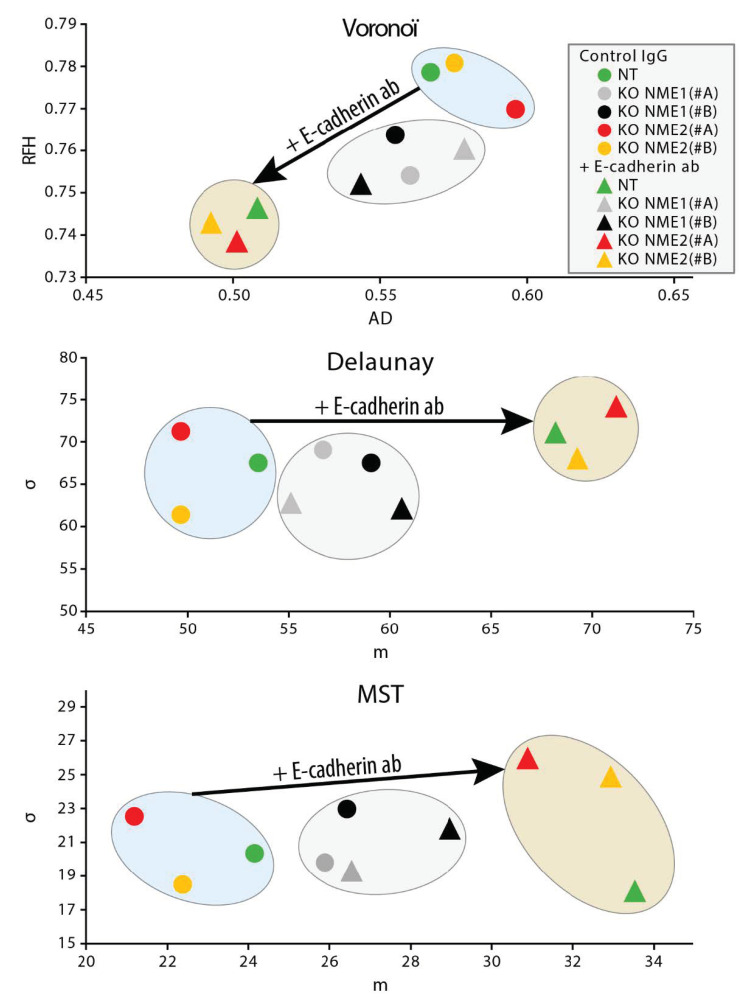
Blocking E-cadherin function does not induce dispersion of NME1-ablated cells. MCF10DCIS.com cells in which the *NME1* gene was inactivated (grey and black symbols), the NME2 gene was inactivated (red and yellow symbols) or non-targeting controls (green symbols) were grown as monolayers in the presence of the E-cadherin-blocking antibody or of an isotype-matched control mouse IgG. Phase-contrast images of the monolayers were analyzed 48 h later by using three methods: Voronoi’s partition (top), Delaunay’s graph (middle) and the minimum spanning tree algorithm (bottom). Two parameters were deduced from each method and were plotted against each other: area disorder (AD) versus roundness factor homogeneity (RFH) for Voronoi’s partition, and average length (m) versus standard deviation (d) for Delaunay’s graph and the minimum spanning tree algorithm. The plots show that growth in the presence of the E-cadherin-blocking antibody (triangles) has a large effect on cell–cell interactions of the non-targeting and NME2-ablated cells when compared to growth in the presence of a control antibody (circles), whereas it has a much smaller effect on the NME1-ablated cells.

Cells were grown as monolayers in the presence of the E-cadherin-blocking antibody or of a isotype-matched control mouse IgG [26], phase-contrast images of the monolayers were analyzed 48 h later for three measures of cell–cell adhesion [26]: Voronoi’s partition, Delaunay’s graph and the minimum spanning tree algorithm. Growing control NT cells or *NME2*-ablated cells in the presence of the antibody resulted in smaller area disorder values for Voronoi’s partition than those in the presence of the control antibody (*p* = 0.011, *p* = 0.00068 and *p* = 0.00154 for NT, NME2(#A) KO, and NME2(#B) KO cells, respectively), and bigger average distance values for Delaunay’s graph (*p* = 0.0275, *p* = 0.0299 and *p* = 0.0078 for NT, NME2(#A) KO, and NME2(#B) KO cells, respectively) and the minimum spanning tree algorithm (*p* = 0.0016, *p* = 0.0052 and *p* = 0.0008 for NT, NME2(#A) KO, and NME2(#B) KO cells, respectively), indicating that blocking E-cadherin function in these cells has a disruptive effect on cell–cell adhesion (Figure 6). Growing *NME1*-ablated cells in the presence of the antibody, by contrast, resulted in no statistically significant differences in these values when compared to the same cells grown in the presence of the control antibody. We conclude from these data that E-cadherin does not contribute significantly to cell–cell adhesion of cells in which *NME1* is inactivated. Thus, E-cadherin-mediated cell–cell adhesion is lost when NME1 is inactivated, but not when NME2 is inactivated.

### 3.6. NME1 Expression Inhibits the Activity of Pro-EMT Signaling Pathways

**Figure 7:**
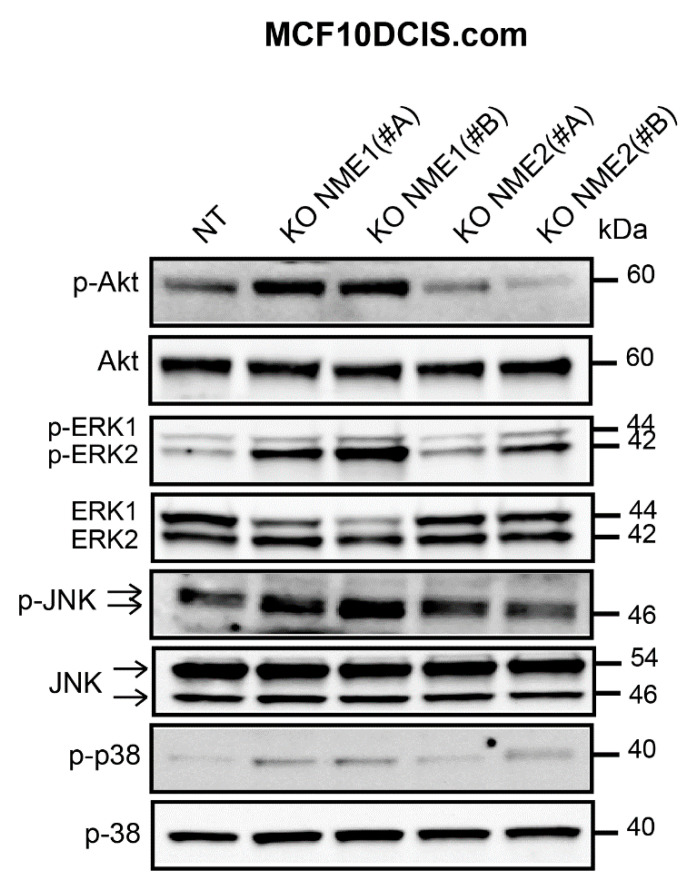
Inactivation of *NME1* stimulates the activity of pro-EMT signaling pathways. Lysates of control (NT) and *NME1* or *NME2*-ablated (KO) MCF10DCIS.com cells were analyzed by Western blotting with antibodies against the phosphorylated forms of Akt (p-Akt), ERK1/ERK2 (p-ERK1/ERK2), JNK (p-JNK) and p38 (p-p38). Molecular weights are indicated in kDa.

**Figure 8:**
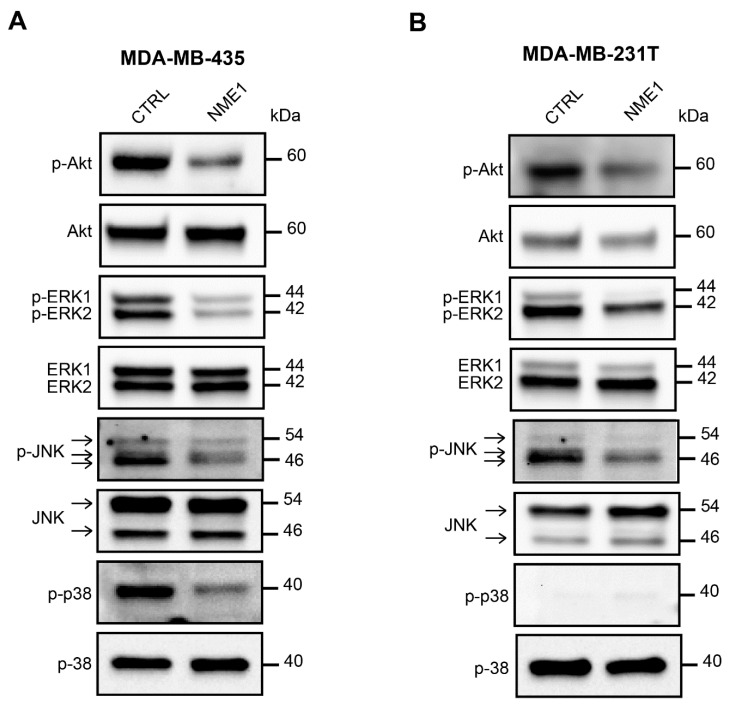
Overexpression of *NME1* inhibits the activity of pro-EMT signaling pathways. (**A**) Lysates of MDA-MB-435 human breast carcinoma cells stably transfected with the empty control vector (CTRL) or with a vector expressing *NME1* (NME1) were analyzed by Western blotting with antibodies against the phosphorylated forms of Akt, and ERK1/2, JNK and p38, as in Figure 7. Molecular weights are indicated in kDa. (**B**) Lysates of MDA-MB-231T human breast carcinoma cells, as in part (**A**).

Mitogenic growth factors promote cancer cell migration and invasion by activating the PI3K/Akt, ERK, JNK, and p38 MAPK signaling pathways and thus inducing EMT. Thus, we determined the level of activation of these pro-EMT pathways according to the modulation of NME1 and NME2 protein levels. When *NME1* was ablated in MCF10DCIS.com cells, Akt was hyperactivated, as indicated by the increased levels of phosphorylated Akt (p-Akt) when compared to the control NT cells (Figure 7). Ablation of *NME1* also induced an increase in the phosphorylation of ERK1/ERK2 and JNK whereas increased phosphorylation of p38 was only moderate (Figure 7). These effects were similar in cells treated with either of the two independent guide RNAs, NME1(#A) and NME1(#B). By contrast, ablation of *NME2* had little or no effect on phosphorylation of these kinases when compared to the control cells (Figure 7). Conversely, overexpression of *NME1* in MDA-MB-435 cells reduced the levels of phosphorylated Akt, ERK, JNK, and p38 (Figure 8A) and similar effects were seen in MDA-MB-231T cells overexpressing *NME1*, except that phosphorylated p38 was detected neither in cells overexpressing *NME1* nor in the control cells (Figure 8B). These data demonstrate a specific role for NME1 as a repressor of essential intracellular signaling pathways involved in EMT.

### 3.7. NME1 Expression Is Reduced in Human Breast Tumor Cell Lines with the Triple-Negative Phenotype

**Figure 9:**
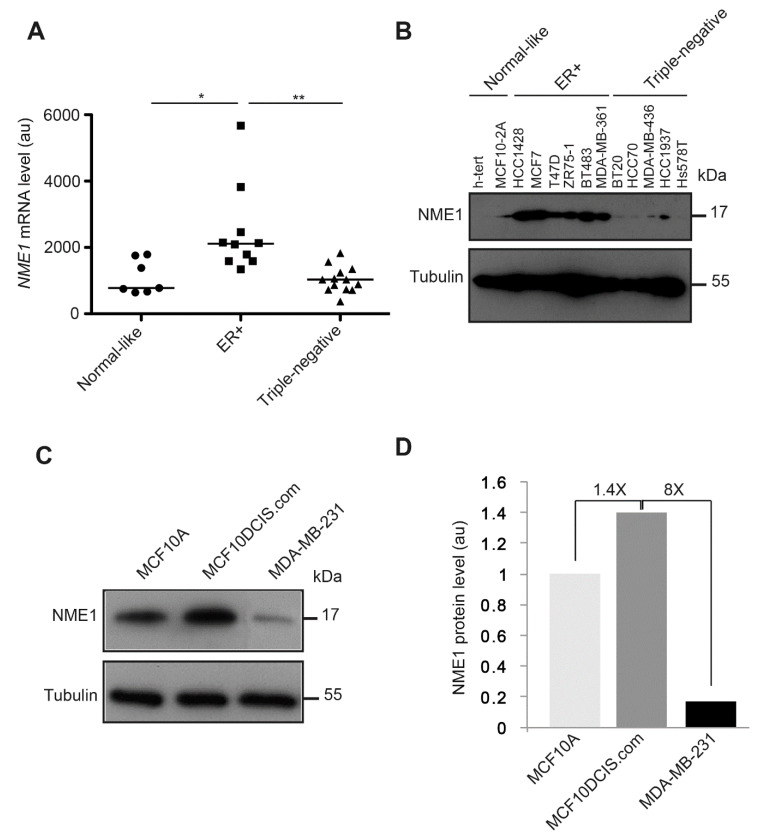
*NME1* expression is reduced in human breast tumor cell lines with the triple-negative phenotype. (**A**) *NME1* mRNA levels were measured by RT–qPCR in normal-like human breast cell lines, in estrogen receptor-positive (ER+) human breast tumor cell lines, and in triple-negative human breast tumor cell lines. Each data point represents one cell line. Three independent analyses were performed for each cell line. Data are expressed as means ± SEM. ** *p* < 0.01, * *p* < 0.05. (**B**) NME1 protein levels were analyzed by Western blotting lysates of normal-like human breast cell lines (h-tert, MCF10-2A), ER+ human breast tumor cell lines (HCC1428, MCF7, T47D, ZR75-1, BT483, and MDA-MB-361) and triple-negative human breast tumor cell lines (BT20, HCC70, MDA-MB-436, HCC1937, and Hs578T). Equal loading was verified by Western blotting for tubulin. Molecular weights are indicated in kDa. (**C**) NME1 protein levels were analyzed by Western blotting lysates of the normal-like human breast cell line MCF10A, the epithelial-like human breast tumor cell line MCF10DCIS.com and the invasive mesenchymal human breast tumor cell line MDA-MB-231. Equal loading was verified by Western blotting for tubulin. Molecular weights are indicated in kDa. (**D**) Quantification of NME1 protein levels as observed in (**C**) in arbitrary unit (au).

To investigate the potential relevance of loss of NME1 for human breast cancer, we analyzed *NME1* transcript levels by RTqPCR and NME1 protein levels by Western blotting of a panel of human breast cell lines according to their normal-like, estrogen receptor (ER)-positive, and triple-negative (hormone receptors negative) status (Figure 9). We observed significantly more *NME1* mRNA in the ER-positive human breast tumor cell lines than in the normal-like cell lines; these levels significantly decreased in the triple-negative human breast tumor cell lines, reaching a similar level to that observed in normal-like cell lines (Figure 9A). These differences between the cell types were even more marked at the protein level: NME1 was barely detected in the normal-like cell lines and in the triple-negative cell lines, whereas it was abundant in all six ER-positive cell lines tested (Figure 9B). Moreover, when compared to the MCF10A normal-like cell line, the MCF10DCIS.com human breast carcinoma cell line had 1.4-fold more NME1 protein, whereas the mesenchymal-type MDA-MB-231 human breast tumor cell line had 8-fold less NME1 protein when compared to MCF10DCIS.com cells (Figure 9C,D). 

### 3.8. NME1 Expression Is Negatively Associated with EMT Markers in Human Cancer

Based on our evidence above that NME1 is a repressor of EMT, we predicted that its expression might be negatively associated with expression of EMT markers in human cancer. To test this prediction, we searched the Molecular Taxonomy of Breast Cancer International Consortium (METABRIC) database—which contains the clinical and biological data for 1904 human breast tumors—looking for relationships between mRNA levels of *NME1* and epithelial and mesenchymal markers (Supplemental Figure S6). We found a positive association of *NME1* mRNA with the mRNAs for the epithelial markers *CDH1* and *KRT18*, which encode E-cadherin and cytokeratin 18, respectively, and a negative association with the mesenchymal marker *VIM,* which encodes vimentin (Supplemental Figure S6A–C). We also observed a negative association of *NME1* mRNA with the mRNAs for EMT drivers including *ZEB1*, *ZEB2*, *SNAI2*, *TWIST1* and *TWIST2* (Supplemental Figure S6D–H). *NME1* mRNA levels were also negatively associated with the overall EMT score (Supplemental Figure S6I,J). We observed similar significant associations between levels of *NME1* mRNA and mRNAs for markers of EMT in the TCGA database of 1100 human breast invasive carcinoma samples, (Supplemental Figure S7A and Table S3). Similar associations were observed for other human cancers in the TCGA database including colorectal adenocarcinoma (382 tumors) (Supplemental Figure S7B and Table S3), liver hepatocellular carcinoma (373 tumors) (Supplemental Figure S7C and Table S3), and prostate adenocarcinoma (498 tumors) (Supplemental Figure S7D and Table S3). In these three types of human cancer, we found a significant positive association between *NME1* mRNA and *KRT18* mRNA, and a negative association between the mRNAs of *NME1*, *VIM* and the EMT drivers *SNAI2* and *ZEB1*. The strongest negative association in these tumors was between *NME1* and *ZEB1* mRNAs, as shown by the very high value of the Spearman’s correlation coefficient. We also found a positive association of *NME1* mRNA with *CDH1* and *KRT18* mRNAs and a negative association with *VIM* mRNA in the human breast tumor cell lines described above (data not shown). These data show that low *NME1* expression associated with high expression of EMT markers is a general feature of human clinical tumor samples.

## 4. Discussion

**Figure 10:**
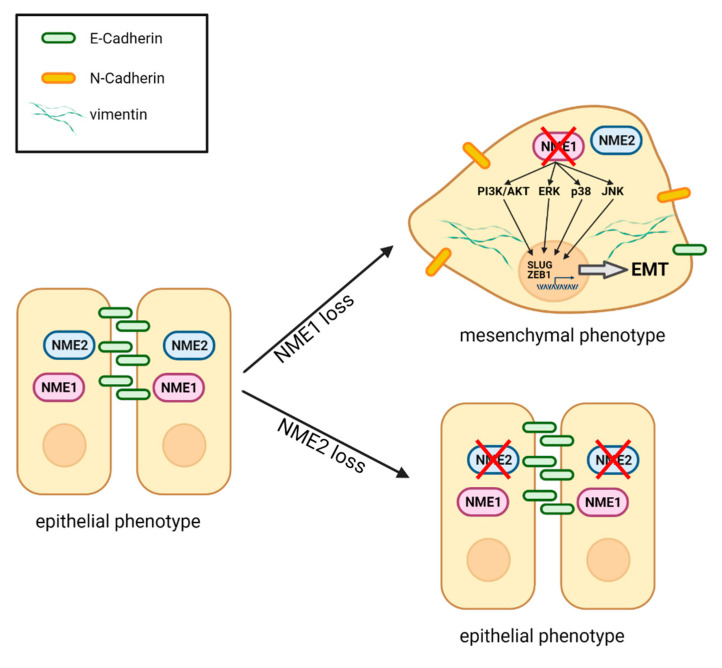
Schematic representation of the effects of loss of NME1 or NME2 on EMT. When NME1 is lost from epithelial cells, the PI3K/AKT, ERK, p38 and JNK pro-EMT signaling pathways are activated. This leads to expression of two key EMT transcription factors SLUG and ZEB1 and launches a partial EMT. The cells lose most E-cadherin from the surface, while they gain N-cadherin on the surface and vimentin in the cytosol. They adopt a more mesenchymal morphology and they lose attachment to neighboring cells. When NME2 is lost, by contrast, there is no effect on the epithelial state of the cells. Figure created with BioRender.com.

EMT is a driver of malignancy important for cancer progression and the initial steps of metastasis. In the present study, we show that the metastasis suppressor NME1 (also called NM23-H1) is a strong inhibitor of EMT in a large variety of cancer cell lines (Figure 10). By contrast, the presence or absence of NME2, which is highly homologous to NME1, has no effect on EMT in the same cancer cells, indicating a highly specific function of NME1 in EMT regulation. Our findings also indicate a key role for NME1 as a repressor of essential intracellular signaling pathways involved in inducing EMT. We show that the absence of NME1 considerably alters the distribution of E-cadherin, a gatekeeper of the epithelial phenotype, shifting it from the plasma membrane to the cytosol and resulting in low levels of this key cell adhesion molecule at the cell surface. We show also that specific inactivation of NME1, but not of NME2, decreases E-cadherin-mediated cell–cell adhesion (Figure 10). Moreover, there is an inverse correlation between expression of NME1 and expression of markers of EMT in human cancer samples.

In two large databases of clinical and biological data on human breast tumors, *NME1* expression was positively associated with expression of epithelial markers and negatively associated with expression of mesenchymal markers and EMT drivers. This was also the case for human colorectal, liver, and prostate cancers. These findings indicate that low levels of *NME1* expression associated with features of EMT is a general characteristic of many human tumors. Consistent with this, NME1 expression is strongly reduced or lost at the invasive front of human colorectal carcinoma [43], which is close to the stroma and where EMT takes place in these tumors. Additionally, we report here that *NME1* mRNA and NME1 protein levels are lowest in cell lines derived from the most aggressive types of human breast tumor, the so-called triple-negative breast tumors, which are in EMT when compared to ER-positive human breast tumor cell lines, which have a more epithelial phenotype. These findings also are consistent with a previous study of human ductal breast carcinomas, in which low or negative E-cadherin expression was associated with lack of ER expression and the triple-negative phenotype [44], which our study shows is also associated with low NME1 expression.

Our findings show that either depletion of *NME1* expression by RNAi or ablation of the *NME1* gene by CRISPR–Cas9 gene editing both induce a partial EMT. Cancer cells that have activated EMT very rarely advance to a fully mesenchymal state; they usually progress to a hybrid, partially epithelial and partially mesenchymal state in which they express a mixture of markers [2,45]. Recent studies revealed that cells in this hybrid state are more stem cell-like or metastatic when compared to true epithelial or mesenchymal cells [46,47,48,49,50]. Consistent with this, co-expression of epithelial and mesenchymal markers correlates with poor clinical outcome for many cancers [46,51] and primary mammary and skin tumors contain multiple tumor cell subpopulations at different stages of EMT and with different metastatic potentials [10]. This latter study also suggested that cells with hybrid phenotypes reach the circulation and form metastases more readily than do cells with a fully mesenchymal phenotype. Another study found that only breast cancer cells undergoing EMT in a non-linear mode had a high metastatic potential [52]. Our data, when considered together with these previous findings, offer new insights to explain why cancer cells without *NME1* are highly metastatic. Why cancer cells in the hybrid state are more metastatic than those with a more clearly mesenchymal phenotype remains an open question.

Whereas numerous studies support the conclusion that loss of NME1 expression correlates with metastasis and poor clinical prognosis in many types of human tumor [24], the role of its highly homologous isoform, NME2, in malignancy is only poorly documented. NME2 is reported to be an inhibitor of cell motility and cell invasion [53,54,55,56,57], one study found enhanced metastasis of *NME2*-depleted lung cancer cells in zebrafish and in nude mice [58], and overexpression of *NME2* in oral squamous carcinoma cells reduced their ability to form lung metastases [59], all of which suggest potentially a similar function to *NME1*. Furthermore, NME2 interacts with plakoglobin (γ-catenin), E-cadherin, and β-catenin and plakoglobin may increase its expression and stability [60]. Our findings show very clearly, however, that, unlike NME1, NME2 is not a repressor of EMT. This raises the question of what exactly is the function of NME2 in cancer. One possible explanation is that both NME1 and NME2 as nucleoside diphosphate kinases might have similar functions at early stages of tumorigenesis to provide nucleoside triphosphates to the proliferating tumor cells but that other, differential functions of these enzymes might be important at later stages when NME1 might be the only isoform to control EMT and metastasis.

Consistent with this hypothesis of biphasic and differential functions of NME1 and NME2, we have previously reported biphasic expression of NME1 in tumor progression, with early overexpression in the primary tumor and less or no expression at the later stages associated with metastatic dissemination [23,43]. One of the most important functions of NME1 is to provide nucleoside triphosphates for nucleic acid synthesis [61]; increased levels of NME1 have been observed in cells induced to proliferate [62,63,64,65,66]. This function might explain why overexpression of NME1 is observed during tumor proliferation at the early stages of tumorigenesis. NME1 expression is subsequently reduced or lost at the invasive front of tumors where the cells proliferate much less and where they induce the EMT and become invasive. The catalytic activity of NME2 is similar to that of NME1 (NME1: 748 U/mg and NME2: 609 U/mg; unpublished data) and this isoform has also been observed to be overexpressed during tumor proliferation [23]. Furthermore, cells transfected with *NME2* proliferate faster than untransfected control cells in culture [59]. These observations together with our evidence that NME2 is not important for EMT are consistent with the hypothesis that both NME1 and NME2 provide nucleoside triphosphates for cancer cell proliferation at early stages of tumorigenesis whereas only NME1 controls EMT and metastasis dissemination.

Our findings show that NME1 and NME2 expression are regulated independently. Our study here of MCF10DCIS.com cells ablated for *NME1* or *NME2*, human brain endothelial cells (hCMEC/D3) silenced for *NME1* or *NME2*, and MDA-MB-435 and MDA-MB-231T cells overexpressing *NME1* show that selective loss or gain of one isoform does not alter the protein level of the other. Thus, the mechanisms that regulate expression of NME1 and NME2 must differ. Studies of *NME1* and *NME2* knockout mice might help to clarify whether the two genes have specific or redundant functions. Single knockout *NME1*^-/-^ mice have defective mammary gland development and their NDPK activity is low in several tissues, suggesting that the loss of NME1 enzyme activity is not compensated by other isoforms, in particular by NME2 [67,68]. Single knockout *NME2*^-/-^ mice, by contrast, have defective KCa3.1 K^+^ channel activity and cytokine production by helper T cells [69]. These findings suggest that NME1 and NME2 have some specific, non-redundant functions, at least for some biological processes.

We do not know yet how NME1 expression is reduced or lost in invasive and metastatic cancer cells. Downregulation of *NME1* expression may be the result of increased methylation of CpG islands at the transcription start site of the *NME1* promoter: it has been reported that inhibitors of DNA methylation can elevate NME1 expression in tumor cells [70], and the protein arginine methyltransferase PRMT5 is directly involved in transcriptional repression of *NME1* [71]. *NME1* expression might be regulated by microRNA: it is reported, for example, that miR-28-3p downregulates *NME1* in colorectal cancer cells [72]. Moreover, several studies suggest that NME1 might be regulated at the protein level: lysosomal cysteine cathepsins, which are proteases with known roles in invasion and metastasis, directly cleave and degrade NME1 [73]; the ubiquitin E3 ligase SCF-FBXO4 interacts with NME1 to mediate its polyubiquitination and subsequent degradation by the proteasome [74]; the hepatitis C virus core protein promotes SUMOylation and degradation of the NME1 protein, as well as its transcriptional downregulation [75] and hepatitis C virus E1 protein also promotes transcriptional downregulation and protein degradation of NME1 [76]. As NME1 is specifically reduced or lost at the invasive front of tumors, the role of the tumor stroma, in particular the role of cancer-associated fibroblasts in regulating NME1 levels remains to be explored. Whether and how NME2 expression might be modulated during cancer progression remains unknown. It would be interesting to determine NME2 protein levels in different subregions of the tumor, i.e., in the highly proliferative central area and at the invasive front.

E-cadherin is considered a ‘gatekeeper’ of the epithelial phenotype [77]. Our findings show that early in the formation of an epithelium, the distribution of E-cadherin is specifically altered by *NME1*-depletion, shifting from the plasma membrane to the cytosol as is characteristic of EMT. The mechanisms involved might include inactivating mutations, epigenetics and transcriptional silencing, altered endocytosis/recycling turnover, and degradation by the proteasome [78]. We observed a decrease in E-cadherin expression levels upon complete *NME1* inactivation by CRISPR–Cas9, whereas expression levels were maintained when *NME1* was incompletely silenced by RNAi. In the latter case, it is unlikely that the change in distribution resulted from a loss of dynamin-dependent endocytosis of E-cadherin induced by *NME1* depletion [79], because this might be expected, on the contrary, to stabilize proteins at the plasma membrane. In agreement with this idea, we showed that loss of *NME1* in human brain endothelial cells did not perturb turnover of the specific endothelial cell–cell adhesion molecule VE-cadherin, a key molecule responsible for the integrity of the endothelium (Supplemental Figures S1F and S8). As an alternative mechanism, increased levels of membrane proteases involved in the cleavage of E-cadherin may be involved in E-cadherin redistribution, including proteases of the ADAM family, whose surface expression can be regulated by dynamin endocytosis [40]. ADAM10 is a candidate for such a protease as its surface expression is regulated by dynamin endocytosis [80] and it was reported to produce soluble E-cadherin fragments that are important in inducing EMT [40]. Consistent with this, ablation of *NME1* upregulates ADAM10 protein levels. Additionally, *NME1* mRNA levels are strongly negatively associated with mRNA levels of *ADAM10* in many human cancers in the TCGA database. This negative association was also observed at the protein level.

In conclusion, we show here that NME1, but not NME2, acts specifically to inhibit EMT and prevent the earliest stages of metastasis. NME1 thus functions as a ‘gatekeeper’ of the epithelial phenotype by providing a barrier against EMT. Reactivation of NME1 expression in tumor cells might thus be a promising avenue to explore for anti-metastatic therapy.

## Data Availability

All data associated with this manuscript will be made available upon request.

## Supplementary Materials

The following are available online at https://www.mdpi.com/article/10.3390/ijms22073718/s1, Supplemental Figure S1. Selective loss or gain of NME1 or NME2 does not alter the protein level of the other isoform. Western blots of (**A**) MCF10DCIS.com human breast carcinoma cells silenced for *NME1* (siNME1, left) or *NME2* (siNME2, right) or treated with a scrambled siRNA (Scr) as a control, showing effective gene silencing. (**B**) Control (NT) and *NME1* or *NME2* knockout (KO) MCF10DCIS.com cells showing effective gene inactivation in two clones (#A and #B) of each gene. (**C**) MDCK epithelial cells stably overexpressing E-cadherin-GFP (MDCK E-cadherin-GFP) silenced for *NME1* (siNME1, left) or *NME2* (siNME2, right) or treated with a scrambled siRNA (Scr), showing effective gene silencing. (**D**) MDA-MB-435 human breast carcinoma cells stably transfected with the empty control vector (CTRL) or with a vector expressing *NME1* (NME1), showing overexpression of NME1. (**E**) MDA-MB-231T human breast carcinoma cells stably transfected with the empty control vector (CTRL) or with a vector expressing *NME1* (NME1), showing overexpression of NME1. (**F**) hCMEC/D3 human brain endothelial cells treated with a scrambled siRNA (Scr) or with siRNAs to silence *NME1* (siNME1) or *NME2* (siNME2), showing effective gene silencing. Equal loading was verified by Western blotting for tubulin (**A**–**E**) or for GAPDH (**F**). Molecular weights are indicated in kDa. Supplemental Figure S2. Genetic validation of *NME1* and *NME2* gene knockout in MCF10DCIS.com clones. Two sites (referred to as A and B) in both the *NME1* and *NME2* genes of MCF10DCIS.com cells were targeted for editing by CRISPR–Cas9. Corresponding targeted sites were sequenced after amplification with appropriate primers (see Table S1). The plot shows the fraction of sequencing reads that aligned with the reference amplicon sequence and for which an insertion or deletion (indel) was observed. Bar colors and x axis labels indicate the amplicon. Labels at the top of the plot indicate the different MCF10DCIS.com cell populations. Parental, MCF10DCIS.com cells not subjected to CRISPR–Cas9 edition; NT, MCF10DCIS.com cells not targeted, i.e., cells subjected to CRISPR–Cas9 edition with a non-targeting control guide; KO NME1(#A) and (#B), MCF10DCIS.com cells subjected to CRISPR–Cas9 edition of *NME1* gene for which two sites A and B were targeted, respectively; KO NME2(#A) and (#B), MCF10DCIS.com cells subjected to CRISPR–Cas9 edition of *NME2* gene for which two sites A and B were targeted, respectively. Supplemental Figure S3: Modulation of NME1 and NME2 levels alters EMT-TF gene expression. (**A**) RT-qPCR analysis of *SNAI1*, *SNAI2*, *TWIST1*, *TWIST2*, *ZEB1* and *ZEB2* mRNA levels in control (NT) and *NME1* or *NME2* knockout (KO) MCF10DCIS.com cells. (**B**) RT-qPCR analysis of *SNAI1*, *SNAI2*, *TWIST1*, *ZEB1* and *ZEB2* mRNA levels in MDA-MB-435 human breast carcinoma cells stably transfected with the empty control vector (CTRL) or with a vector expressing *NME1* (NME1). (**C**) RT-qPCR analysis of *SNAI1*, *SNAI2*, *ZEB1* and *ZEB2* mRNA levels in MDA-MB-231T human breast carcinoma cells stably transfected with the empty control vector (CTRL) or with a vector expressing *NME1* (NME1). All data were normalized to the levels of PGK1 and TBP mRNAs. An average of 9 biological replicates from 3 independent experiments are shown; error bars indicate SEM. * *p* < 0.05; ** *p* < 0.01; *** *p* < 0.001, **** *p* < 0.0001. Supplemental Figure S4: Loss of NME1 induces cleavage of E-cadherin and upregulation of ADAM10. Left: Western blot of MDCK epithelial cells stably overexpressing E-cadherin-GFP (MDCK E-cadherin-GFP) and treated with a control siRNA (Scr) or siRNAs to silence *NME1* (siNME1) or *NME2* (siNME2), showing E-cadherin-GFP, endogenous E-cadherin and E-cadherin fragments. The results of two independent experiments (Exp1 and Exp2) are shown. Right: Quantification of the intensities of the E-cadherin fragments in NME1- and NME2-silenced cells relative to the Scr control. The higher fragment, with an intensity approximately proportional to the undegraded E-cadherin signal, was not included in the analysis because its high intensity masked the signals from the other cleavage products. (**B**) Western blot of MDCK E-cadherin-GFP cells treated with a control siRNA (Scr) or with an siRNA to silence *NME1* (siNME1) and probed with an antibody against ADAM10. The results of three independent experiments are shown. (**C**) Western blots of control (NT) and knockout (KO) MCF10DCIS.com cells targeted with two different guide RNAs for each gene (NME1#A, NME1#B, NME2#A, NME2#B) and probed with an antibody against ADAM10. (**D**) MDA-MB-231T human breast carcinoma cells stably transfected with the empty control vector (CTRL) or with a vector expressing *NME1* (NME1) were analyzed by Western blot with an antibody against ADAM10. In all cases, equal loading was verified by Western blotting for tubulin and molecular weights are indicated in kDa. Supplemental Figure S5: Association between NME1 and ADAM10 mRNA and protein levels in invasive breast carcinoma. Data on NME1 and ADAM10 mRNA and protein levels in invasive breast carcinoma were retrieved from the TCGA database and analyzed for their correlation. (**A**) Protein levels of NME1 and ADAM10 correlated negatively. (**B**) mRNA and protein levels of NME1 correlated positively. (**C**) mRNA and protein levels of ADAM10 correlated positively. The Spearman’s rank correlation coefficients are indicated on each plot. Supplemental Figure S6. Association between expression of *NME1* and regulators of EMT in breast cancer. Data on the mRNA levels of *NME1* and various epithelial and mesenchymal marker genes and EMT driver genes were retrieved from the METABRIC database (1904 human breast tumors) and analyzed for their correlations. Epithelial markers, *CDH1* (**A**) and *KRT18* (**B**); mesenchymal marker *VIM* (**C**); EMT drivers, *ZEB1* (**D**), *ZEB2* (**E**), *SNAI2* (**F**), *TWIST1* (**G**), *TWIST2* (**H**), and EMT score (**I**). Correlation coefficients are summarized in (**J**). Supplemental Figure S7. Association between expression of *NME1* and markers of EMT in various carcinomas. Data on mRNA levels of *NME1* and the EMT marker genes *KRT18, VIM, SNAI2* and *ZEB1* were retrieved from the TCGA database and analyzed for their correlations. (**A**) Breast invasive carcinoma. (**B**) Colorectal adenocarcinoma. (**C**) Liver hepatocellular carcinoma. (**D**) Prostate adenocarcinoma. See also Supplementary Table S3. Supplemental Figure S8. Loss of *NME1* does not perturb brain endothelial cell monolayer permeability. FITC-dextran vascular permeability was measured on 3-day-old human brain endothelial cells (hCMEC/D3) silenced for *NME1* (siNME1) or *NME2* (siNME2), or treated with a scrambled siRNA (Scr) as a control. Permeability is expressed as fold increase with respect to untreated cells. Three independent experiments were performed. Data are expressed as means ± SEM. VEGF was used as a control known to increase vascular permeability. VEGF promotes dynamin-dependent clathrin-mediated endocytosis of VE-cadherin, an endothelial specific cell–cell adhesion molecule, thereby disrupting the endothelial barrier and inducing an increase in the endothelial permeability. Silencing of α-adaptin (si α-adaptin), a major component of the AP2 molecule involved in clathrin-mediated endocytosis, prevented the VEGF-mediated increase in endothelial permeability, as expected. Conversely, depletion of *NME1* or *NME2* did not modify VEGF-driven endothelial permeability, indicating that NME1 and NME2 does not control VE-cadherin turnover in response to VEGF in human brain endothelial cells. Table S1. Nextera XT adapter overhangs sequences and locus specific primer sequences used for CRISPR–Cas9 editing of *NME1* and *NME2* genes. For both *NME1* and *NME2* genes, two sites (referred to as A and B) were targeted for editing by CRISPR–Cas9 in MCF10DCIS.com cells. Corresponding targeted sites were sequenced after amplification with appropriate primers. Table S2: Association between expression of *NME1* and *ADAM10* in various tumors in the human TCGA database. Data on mRNA levels of *NME1* and *ADAM10* in fifteen types of tumor were retrieved from the TCGA database and analyzed for their correlations. Table S3. Association between expression of *NME1* and markers of EMT in various carcinomas. Data on mRNA levels of *NME1* and the EMT marker genes *CDH1, KRT18, CDH2, VIM, SNAI1, SNAI2, ZEB1, ZEB2, TWIST1* and *TWIST2* in breast invasive carcinoma, colorectal adenocarcinoma, liver hepatocellular carcinoma, and prostate adenocarcinoma were retrieved from the TCGA database and analyzed for their correlations.
