# Supplementary material for: Loss of the Metastasis Suppressor NME1, But Not of Its Highly Related Isoform NME2, Induces a Hybrid Epithelial–Mesenchymal State in Cancer Cells
Source: Int J Mol Sci. 2021 Apr 2;22(7):3718. doi: 10.3390/ijms22073718 (PMC8038181; doi:10.3390/ijms22073718)
Supplement: Supplementary file 1 [file ijms-22-03718-s001.pdf]

International Journal of
Molecular Sciences

MDPI

Supplemental Figure 1

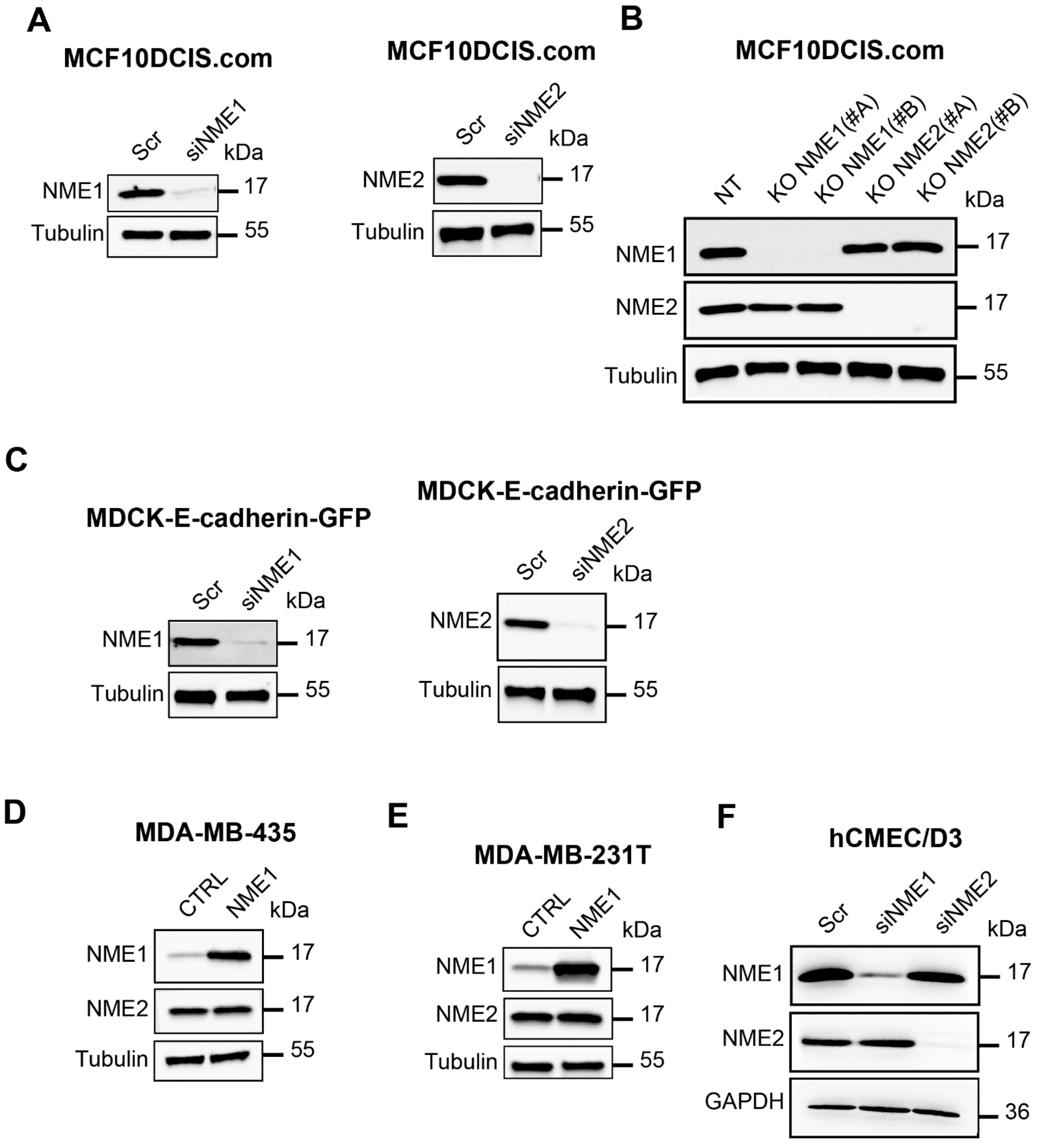

Supplemental Figure 1. Selective loss or gain of NME1 or NME2 does not alter the protein level of the other isoform. Western blots of **A)** MCF10DCIS.com human breast carcinoma cells silenced for *NME1* (siNME1, left) or *NME2* (siNME2, right) or treated with a scrambled siRNA (Scr) as a control, showing effective gene silencing. **B)** Control (NT) and *NME1* or *NME2* knockout (KO) MCF10DCIS.com cells showing effective gene inactivation in two clones (#A and #B) of each gene. **C)** MDCK epithelial cells stably overexpressing E-cadherin-GFP (MDCK E-cadherin-GFP) silenced for *NME1* (siNME1, left) or *NME2* (siNME2, right) or treated with a scrambled siRNA (Scr), showing effective gene silencing. **D)** MDA-MB-435 human breast carcinoma

cells stably transfected with the empty control vector (CTRL) or with a vector expressing *NME1* (NME1), showing overexpression of NME1. **E)** MDA-MB-231T human breast carcinoma cells stably transfected with the empty control vector (CTRL) or with a vector expressing *NME1* (NME1), showing overexpression of NME1. **F)** hCMEC/D3 human brain endothelial cells treated with a scrambled siRNA (Scr) or with siRNAs to silence *NME1* (siNME1) or *NME2* (siNME2), showing effective gene silencing. Equal loading was verified by western blotting for tubulin (A–E) or for GAPDH (F). Molecular weights are indicated in kDa.

Supplemental Figure 2

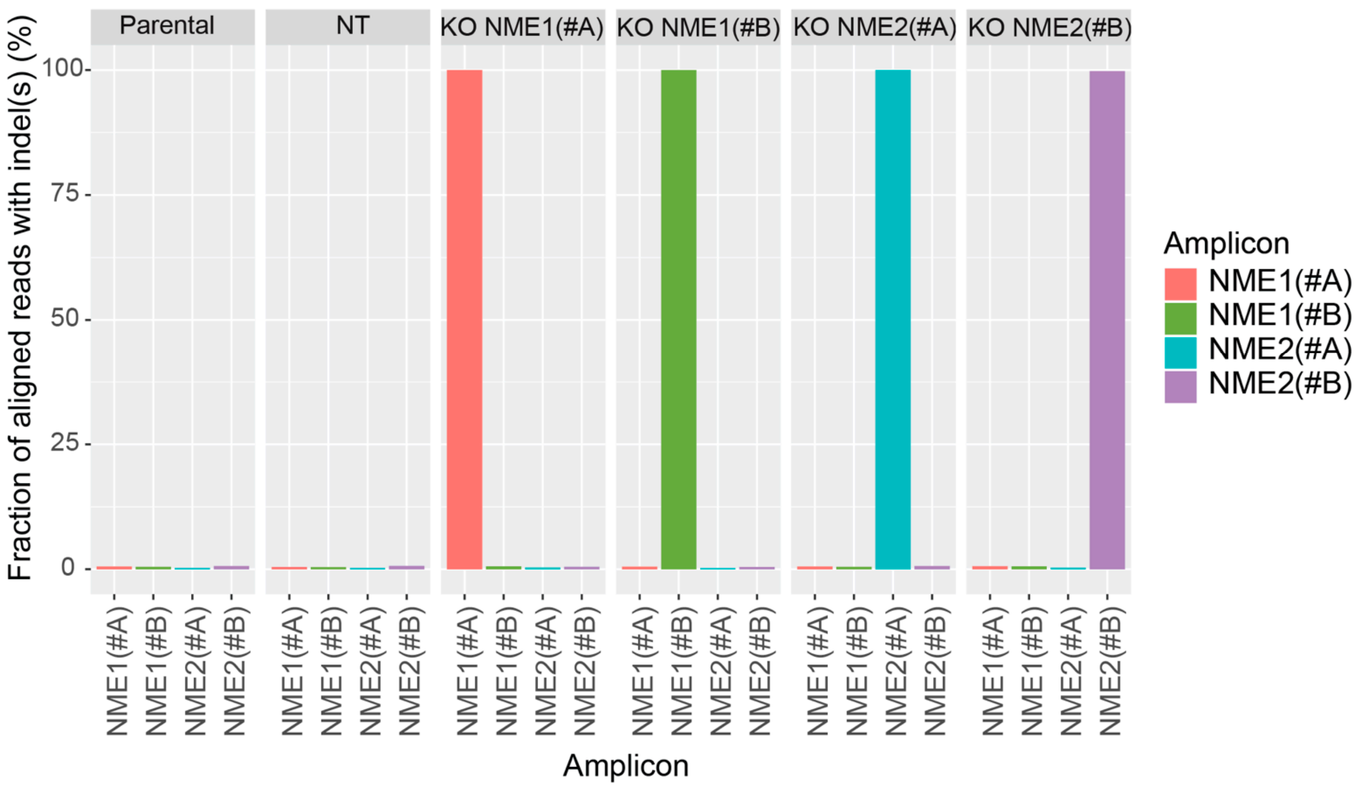

Supplemental Figure 2. Genetic validation of *NME1* and *NME2* gene knockout in MCF10DCIS.com clones. Two sites (referred to as A and B) in both the *NME1* and *NME2* genes of MCF10DCIS.com cells were targeted for editing by CRISPR–Cas9. Corresponding targeted sites were sequenced after amplification with appropriate primers (see Table S1). The plot shows the fraction of sequencing reads that aligned with the reference amplicon sequence and for which an insertion or deletion (indel) was observed. Bar colors and x axis labels indicate the amplicon. Labels at the top of the plot indicate the different MCF10DCIS.com cell populations. Parental, MCF10DCIS.com cells not subjected to CRISPR–Cas9 edition; NT, MCF10DCIS.com cells not targeted, *i.e.* cells subjected to CRISPR–Cas9 edition with no guide; KO NME1(#A) and (#B), MCF10DCIS.com cells subjected to CRISPR–Cas9 edition of *NME1* gene for which two sites A and B were targeted, respectively; KO NME2(#A) and (#B), MCF10DCIS.com cells subjected to CRISPR–Cas9 edition of *NME2* gene for which two sites A and B were targeted, respectively.

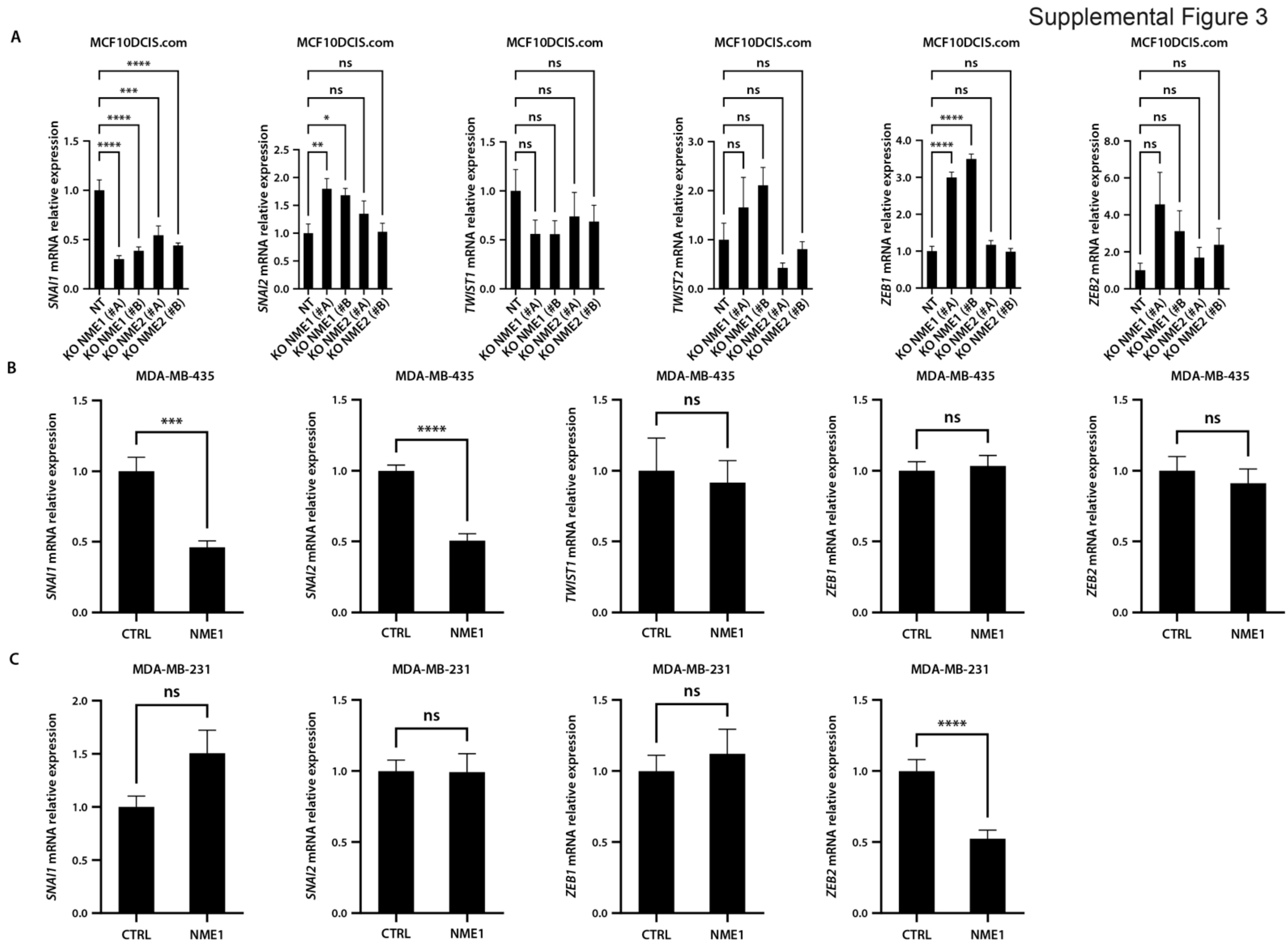

Supplemental Figure 3: Modulation of NME1 and NME2 levels alters EMT-TF gene expression. A) RT-qPCR analysis of *SNAI1*, *SNAI2*, *TWIST1*, *TWIST2*, *ZEB1* and *ZEB2* mRNA levels in control (NT) and *NME1* or *NME2* knockout (KO) MCF10DCIS.com cells. **B)** RT-qPCR analysis of *SNAI1*, *SNAI2*, *TWIST1*, *ZEB1* and *ZEB2* mRNA levels in MDA-MB-435 human breast carcinoma cells stably transfected with the empty control vector (CTRL) or with a vector expressing *NME1* (NME1). **C)** RT-qPCR analysis of *SNAI1*, *SNAI2*, *ZEB1* and *ZEB2* mRNA levels in MDA-MB-231T human breast carcinoma cells stably transfected with the empty control vector (CTRL) or with a vector expressing *NME1* (NME1). All data were normalized to the levels of PGK1 and TBP mRNAs. An average of 9 biological replicates from 3 independent experiments are shown; error bars indicate SEM. *$P < 0.05$; **$P < 0.01$; ***$P < 0.001$, ****$P < 0.0001$.

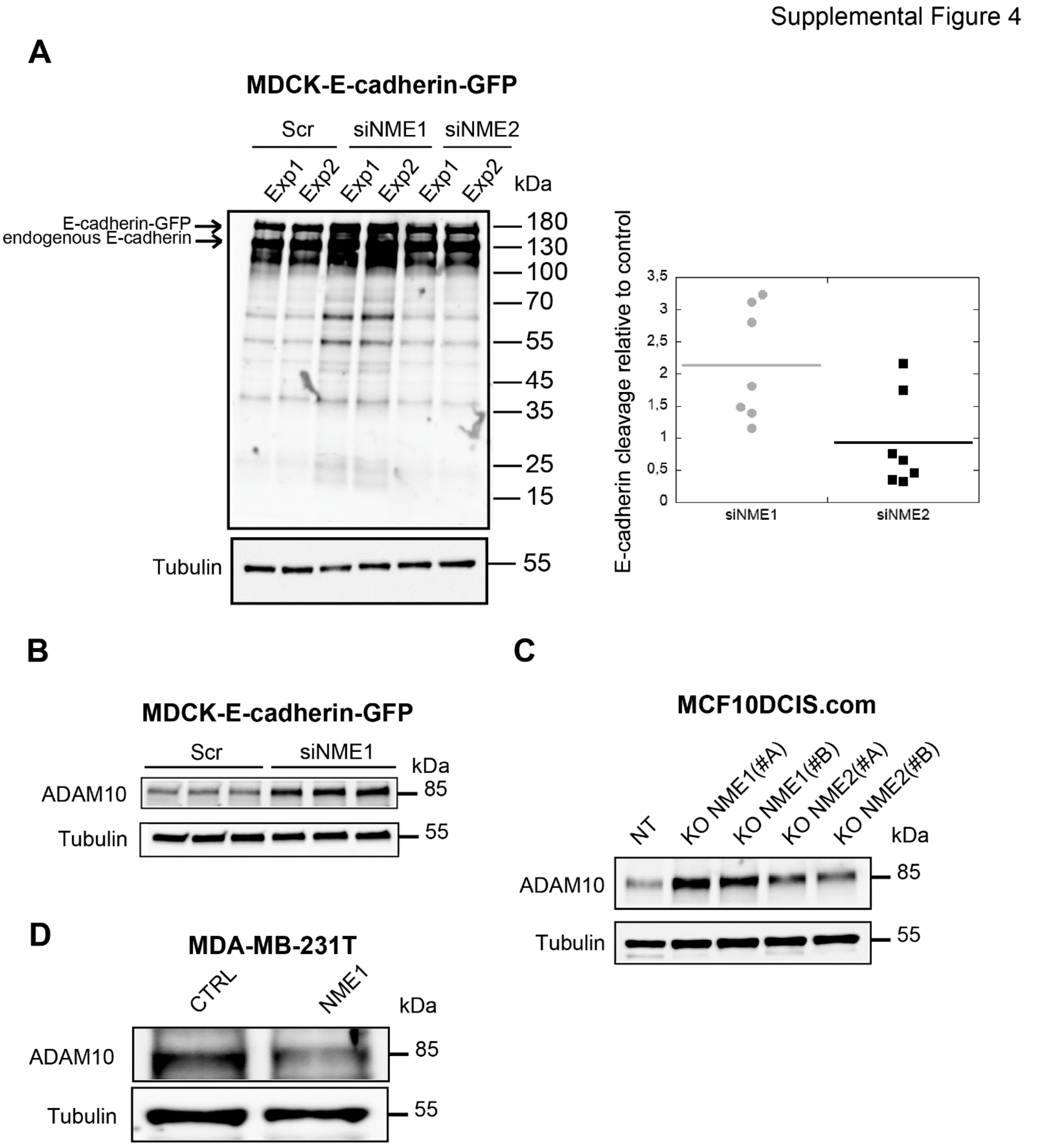

Supplemental Figure 4: Loss of NME1 induces cleavage of E-cadherin and upregulation of ADAM10. Left: western blot of MDCK epithelial cells stably overexpressing E-cadherin-GFP (MDCK E-cadherin-GFP) and treated with a control siRNA (Scr) or siRNAs to silence *NME1* (siNME1) or *NME2* (siNME2), showing E-cadherin-GFP, endogenous E-cadherin and E-cadherin fragments. The results of two independent experiments (Exp1 and Exp2) are shown. Right: Quantification of the intensities of the E-cadherin fragments in NME1- and NME2-silenced cells relative to the Scr control. The higher fragment, with an intensity approximately proportional to the undegraded E-cadherin signal, was not included in the analysis because its high intensity masked the signals from the other cleavage products. **B)** Western blot of MDCK E-cadherin-GFP cells treated with a control siRNA (Scr) or with an siRNA to silence *NME1* (siNME1) and probed with an antibody against ADAM10. The results of three independent experiments are shown. **C)** Western blots of control (NT) and knockout (KO) MCF10DCIS.com cells targeted with

two different guide RNAs for each gene (NME1#A, NME1#B, NME2#A, NME2#B) and probed with an antibody against ADAM10. **D)** MDA-MB-231T human breast carcinoma cells stably transfected with the empty control vector (CTRL) or with a vector expressing *NME1* (NME1) were analyzed by western blot with an antibody against ADAM10. In all cases, equal loading was verified by western blotting for tubulin and molecular weights are indicated in kDa.

Supplemental Figure 5

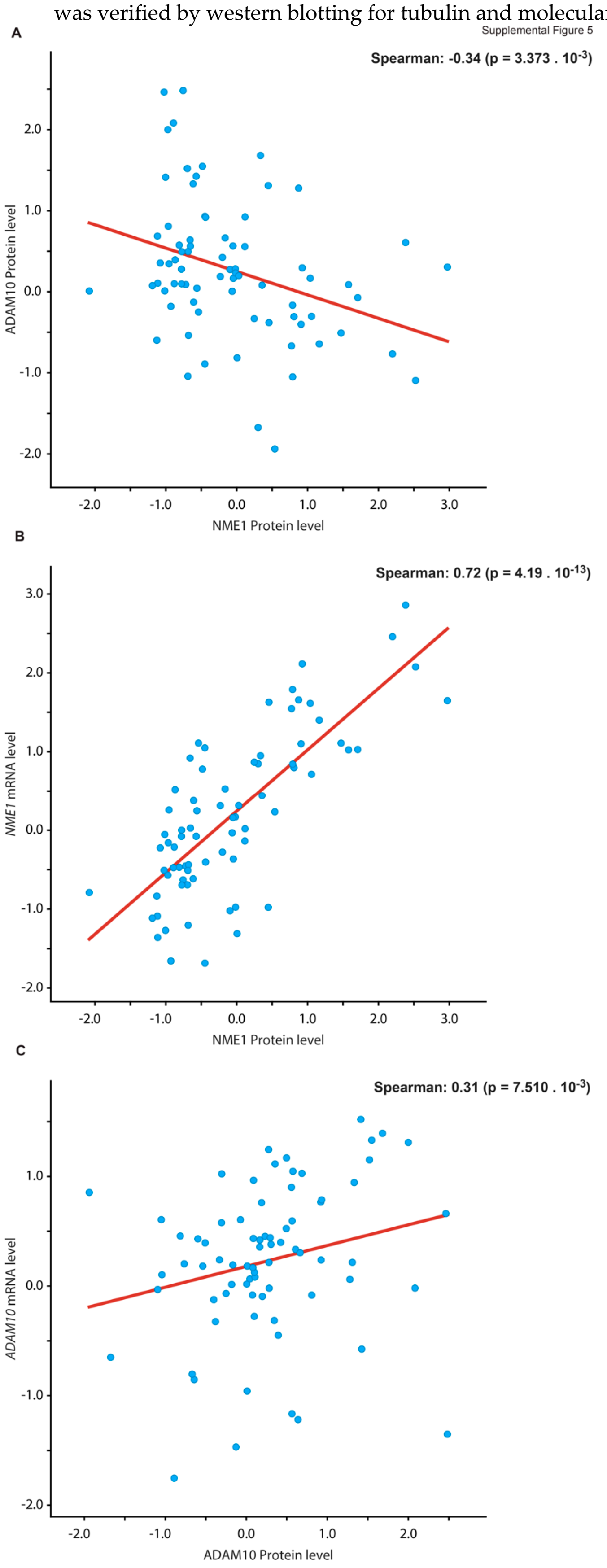

Supplemental Figure 5: Association between NME1 and ADAM10 mRNA and protein levels in invasive breast carcinoma. Data on NME1 and ADAM10 mRNA and protein levels in invasive breast carcinoma were retrieved from the TCGA database and analyzed for their correlation. **A)** mRNA and protein levels of ADAM10 correlated positively. **B)** mRNA and protein levels of NME1 correlated positively. **C)** Protein levels of NME1 and ADAM10 correlated negatively. The Spearman's rank correlation coefficients are indicated on each plot.

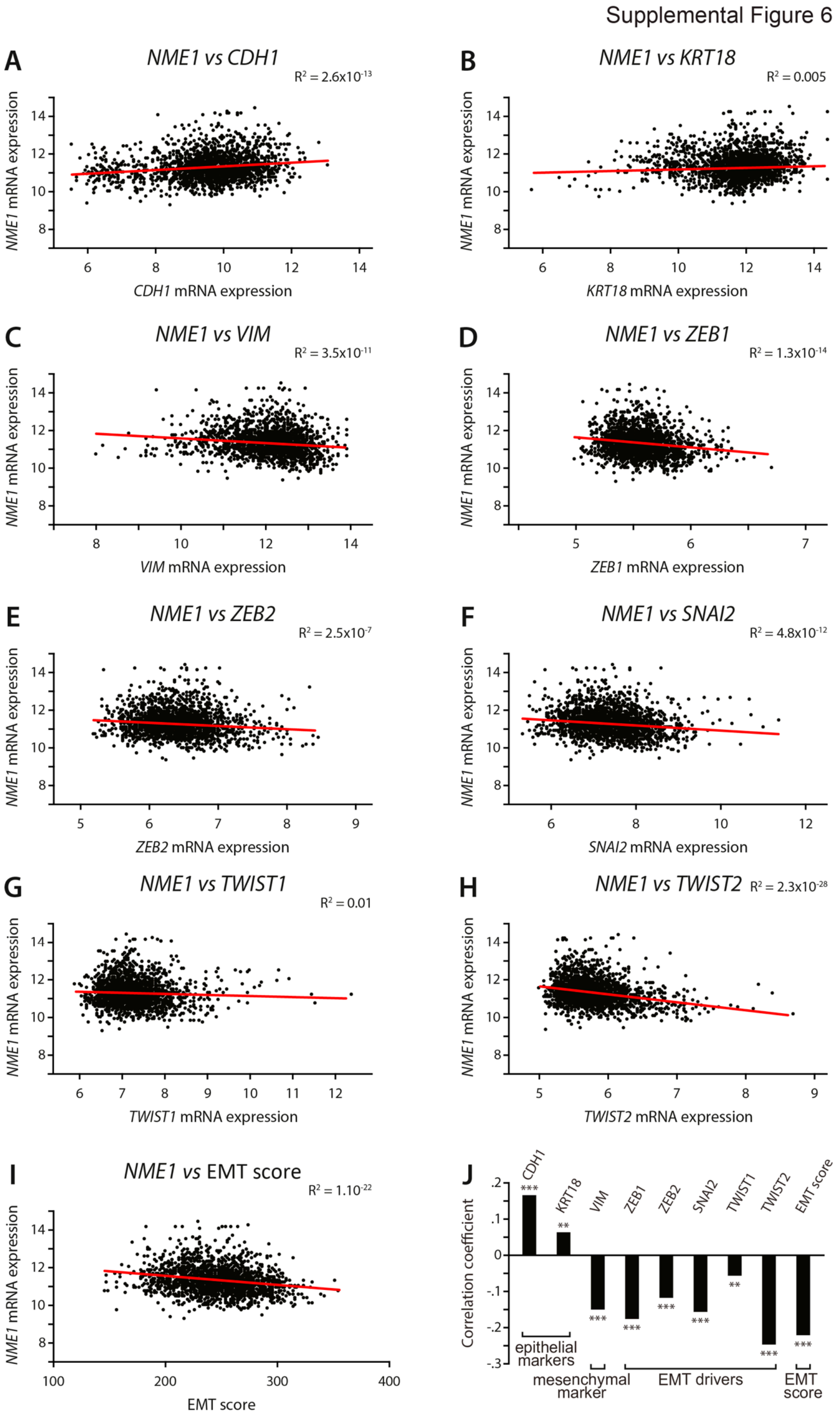

Supplemental Figure 6. Association between expression of *NME1* and regulators of EMT in breast cancer. Data on the mRNA levels of *NME1* and various epithelial and mesenchymal marker genes and EMT driver genes were retrieved

from the METABRIC database (1904 human breast tumors) and analyzed for their correlations. Epithelial markers, *CDH1* **(A)** and *KRT18* **(B)**; mesenchymal marker *VIM* **(C)**; EMT drivers, *ZEB1* **(D)**, *ZEB2* **(E)**, *SNAI2* **(F)**, *TWIST1* **(G)**, *TWIST2* **(H)**, and the EMT score **(I)**. Correlation coefficients are summarized in **(J)**.

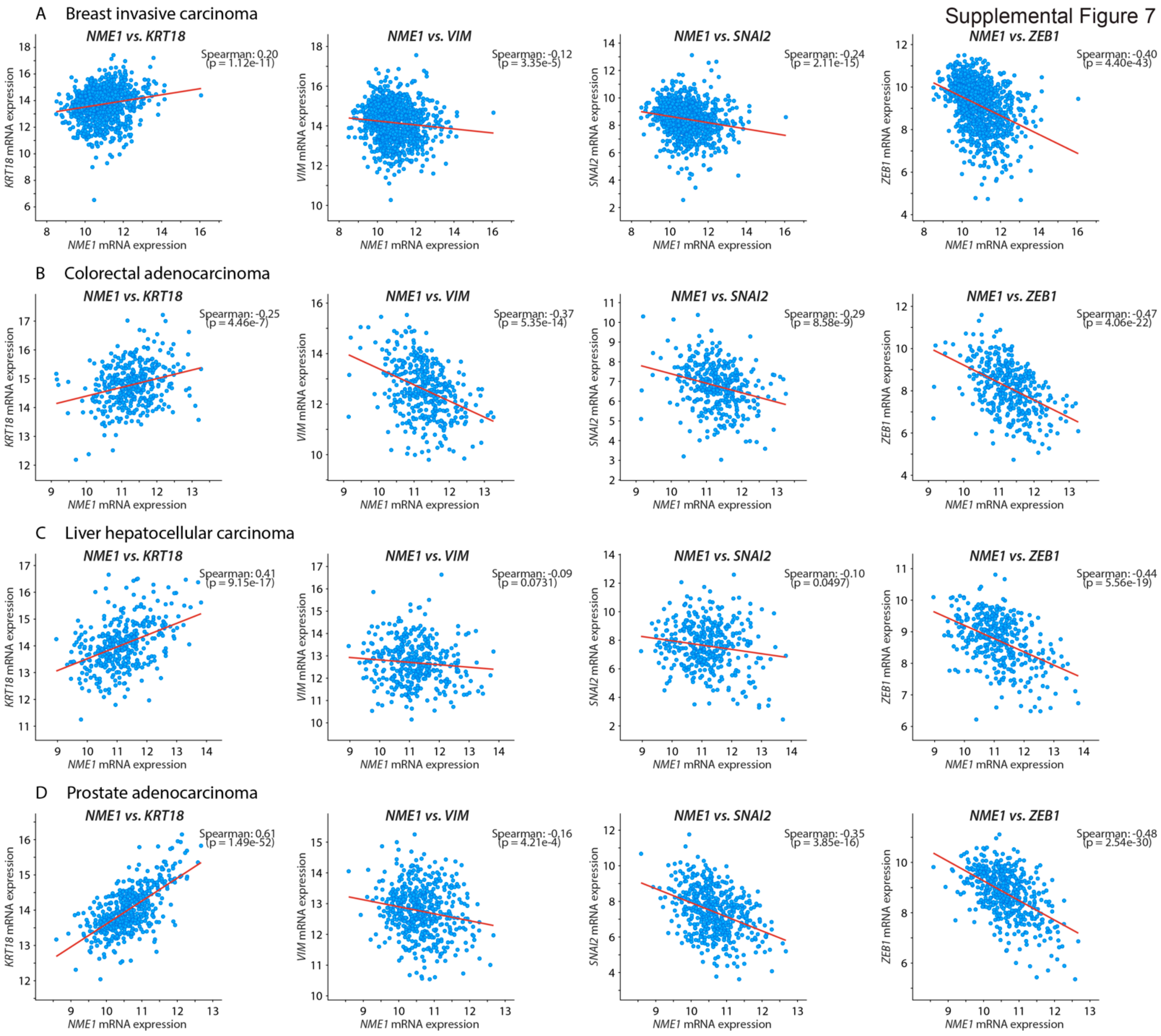

Supplemental Figure 7. Association between expression of *NME1* and markers of EMT in various carcinomas. Data on mRNA levels of *NME1* and the EMT marker genes *KRT18, VIM, SNAI2* and *ZEB1* were retrieved from the TCGA database and analyzed for their correlations. **(A)** Breast invasive carcinoma. **(B)** Colorectal adenocarcinoma. **(C)** Liver hepatocellular carcinoma. **(D)** Prostate adenocarcinoma. See also Supplementary Table S2.

Supplemental Figure 8

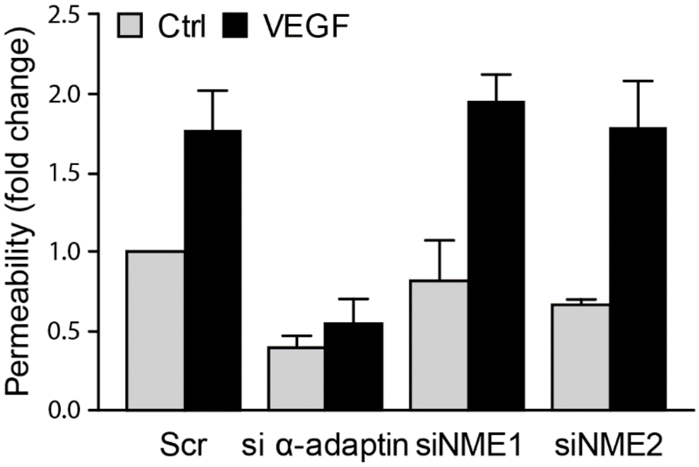

Supplemental Figure 8. Loss of *NME1* does not perturb brain endothelial cell monolayer permeability. FITC-dextran vascular permeability was measured on 3-day-old human brain endothelial cells (hCMEC/D3) silenced for *NME1* (siNME1) or *NME2* (siNME2), or treated with a scrambled siRNA (Scr) as a control. Permeability is expressed as fold increase with respect to untreated cells. Three independent experiments were performed. Data are expressed as means ± SEM. VEGF was used as a control known to increase vascular permeability. VEGF promotes dynamin-dependent clathrin-mediated endocytosis of VE-cadherin, an endothelial specific cell-cell adhesion molecule, thereby disrupting the endothelial barrier and inducing an increase of the endothelial permeability. Silencing of α-adaptin (si α-adaptin), a major component of the AP2 molecule involved in clathrin-mediated endocytosis, prevented the VEGF-mediated increase in endothelial permeability, as expected. Conversely, depletion of *NME1* or *NME2* did not modify VEGF-driven endothelial permeability, indicating that NME1 and NME2 does not control VE-cadherin turnover in response to VEGF in human brain endothelial cells.

**Primer name**	**Adapter sequence**	**Locus specific primer sequence**
NME1A-Fwd	TCGTCGGCAGCGTCAGATGTGTATAAGAGACAG	AATAGTTGCCAGATTTTCTGCTGT
NME1A-Rev	GTCTCGTGGGCTCGGAGATGTGTATAAGAGACAG	GGGAAAAATACCAAAATCTCACCT
NME1B-Fwd	TCGTCGGCAGCGTCAGATGTGTATAAGAGACAG	CAGTGTGGAGAATGAATTGGGTTA
NME1B-Rev	GTCTCGTGGGCTCGGAGATGTGTATAAGAGACAG	AGTATCCCACACAGGCACACTC
NME2A-Fwd	TCGTCGGCAGCGTCAGATGTGTATAAGAGACAG	GCGTGGTGGGGGAGGAG
NME2A-Rev	GTCTCGTGGGCTCGGAGATGTGTATAAGAGACAG	GGAGACGGGGGCGAGTTACC
NME2B-Fwd	TCGTCGGCAGCGTCAGATGTGTATAAGAGACAG	GACTTGCTAATGGGAGGTTCAGAG
NME2B-Rev	GTCTCGTGGGCTCGGAGATGTGTATAAGAGACAG	CAAAGAACACTGAGCACTTTTTCC

Table S1

Table S1. Nextera XT adapter overhangs sequences and locus specific primer sequences used for CRISPR–Cas9 editing of *NME1* and *NME2* genes. For both *NME1* and *NME2* genes, two sites (referred to as A and B) were targeted for editing by CRISPR–Cas9 in MCF10DCIS.com cells. Corresponding targeted sites were sequenced after amplification with appropriate primers.

Tumor type	Sample size (n)	Spearman's correlation	p-value
Breast invasive carcinoma	1100	-0.350	$4.86.10^{-33}$
Colorectal carcinoma	382	-0.327	$6.12.10^{-11}$
Cervical squamous cell carcinoma	297	-0.283	$7.99.10^{-7}$
Esophageal carcinoma	185	-0.293	$5.098.10^{-5}$
Head and neck squamous cell carcinoma	522	-0.438	$7.75.10^{-26}$
Kidney renal clear cell carcinoma	534	-0.456	$8.92.10^{-29}$
Liver hepatocellular carcinoma	373	-0.453	$2.76.10^{-20}$
Lung adenocarcinoma	517	-0.132	$2.733.10^{-3}$
Ovarian serous cystadenocarcinoma	307	-0.403	$2.18.10^{-13}$
Pancreatic adenocarcinoma	179	-0.214	$3.984.10^{-3}$
Prostate adenocarcinoma	498	-0.542	$2.00.10^{-39}$
Testicular germ cell cancer	156	-0.388	$5.62.10^{-7}$
Thyroid carcinoma	509	-0.696	$7.21.10^{-75}$
Uterine corpus endometrial carcinoma	177	-0.460	$1.23.10^{-10}$
Skin cutaneous melanoma	472	-0.243	$9.02.10^{-8}$

Table S2

Table S2: Association between expression of *NME1* and *ADAM10* in various tumors in the human TCGA database. Data on mRNA levels of *NME1* and *ADAM10* in fifteen types of tumor were retrieved from the TCGA database and analyzed for their correlations.

Correlated gene	Description	Spearman's correlation	p-value
NME1, breast invasive carcinoma, n=1100			
CDH1	E-cadherin	0.128	$2.002.10^{-5}$
KRT18	Cytokeratin-18	0.203	$1.12.10^{-11}$
CDH2	N-cadherin	-0.0172	0.569
VIM	Vimentin	-0.125	$3.351.10^{-5}$
SNAI1	Snail	0.103	$6.163.10^{-4}$
SNAI2	Slug	-0.236	$2.11.10^{-15}$
ZEB1	ZEB1	-0.398	$4.40.10^{-43}$
ZEB2	ZEB2	-0.372	$2.29.10^{-37}$
TWIST1	TWIST1	-0.0324	0.282
TWIST2	TWIST2	-0.148	$8.20.10^{-7}$
NME1, colorectal adenocarcinoma, n=382			
CDH1	E-cadherin	-0.0323	0.529
KRT18	Cytokeratin-18	0.255	$4.46.10^{-7}$
CDH2	N-cadherin	-0.351	$1.50.10^{-12}$
VIM	Vimentin	-0.372	$5.35.10^{-14}$
SNAI1	Snail	0.0992	0.0526
SNAI2	Slug	-0.289	$8.58.10^{-9}$
ZEB1	ZEB1	-0.467	$4.06.10^{-22}$
ZEB2	ZEB2	-0.462	$1.25.10^{-21}$
TWIST1	TWIST1	-0.219	$1.523.10^{-5}$
TWIST2	TWIST2	-0.296	$2.84.10^{-9}$
NME1, liver hepatocellular carcinoma, n=373			
CDH1	E-cadherin	-0.352	$2.43.10^{-12}$
KRT18	Cytokeratin-18	0.413	$9.15.10^{-17}$
CDH2	N-cadherin	-0.189	$2.429.10^{-4}$
VIM	Vimentin	-0.0929	0.0731
SNAI1	Snail	-0.180	$4.914.10^{-4}$
SNAI2	Slug	-0.102	0.0497
ZEB1	ZEB1	-0.439	$5.56.10^{-19}$
ZEB2	ZEB2	-0.371	$1.32.10^{-13}$
TWIST1	TWIST1	-0.0343	0.509
TWIST2	TWIST2	-0.0819	0.114
NME1, prostate adenocarcinoma, n=498			
CDH1	E-cadherin	-0.225	$3.75.10^{-7}$
KRT18	Cytokeratin-18	0.612	$1.49.10^{-52}$
CDH2	N-cadherin	-0.234	$1.29.10^{-7}$
VIM	Vimentin	-0.157	$4.217.10^{-4}$

SNAI1	Snail	-0.140	$1.687.10^{-3}$
SNAI2	Slug	-0.354	$3.85.10^{-16}$
ZEB1	ZEB1	-0.482	$2.54.10^{-30}$
ZEB2	ZEB2	-0.595	$5.18.10^{-49}$
TWIST1	TWIST1	0.192	$1.618.10^{-5}$
TWIST2	TWIST2	-0.177	$7.343.10^{-5}$

Table S3

Table S3. Association between expression of *NME1* and markers of EMT in various carcinomas. Data on mRNA levels of *NME1* and the EMT marker genes *CDH1, KRT18, CDH2, VIM, SNAI1, SNAI2, ZEB1, ZEB2, TWIST1* and *TWIST2* in breast invasive carcinoma, colorectal adenocarcinoma, liver hepatocellular carcinoma, and prostate adenocarcinoma were retrieved from the TCGA database and analyzed for their correlations.
